# Metal specificity of cyanobacterial nickel-responsive repressor InrS: cells maintain zinc and copper below the detection threshold for InrS

**DOI:** 10.1111/mmi.12594

**Published:** 2014-04-14

**Authors:** Andrew W Foster, Rafael Pernil, Carl J Patterson, Nigel J Robinson

**Affiliations:** 1 Department of Chemistry, School of Biological and Biomedical Sciences, Durham UniversityDurham, DH1 3LE, UK

## Abstract

InrS is a Ni(II)-responsive, CsoR/RcnR-like, DNA-binding transcriptional repressor of the *nrsD* gene, but the Ni(II) co-ordination sphere of InrS is unlike Ni(II)-RcnR. We show that copper and Zn(II) also bind tightly to InrS and *in vitro* these ions also impair InrS binding to the *nrsD* operator-promoter. InrS does not respond to Zn(II) (or copper) *in vivo* after 48 h, when Zn(II) sensor ZiaR responds, but InrS transiently responds (1 h) to both metals. InrS conserves only one (of two) second co-ordination shell residues of CsoR (Glu98 in InrS). The allosteric mechanism of InrS is distinct from Cu(I)-CsoR and conservation of deduced second shell residues better predicts metal specificity than do the metal ligands. The allosteric mechanism of InrS permits greater promiscuity *in vitro* than CsoR. The factors dictating metal-selectivity *in vivo* are that *K*_Ni__(__II__)_ and Δ*G*_C_^Ni^^(^^II^^)-^^InrS^^·^^DNA^ are sufficiently high, relative to other metal sensors, for InrS to detect Ni(II), while the equivalent parameters for copper may be insufficient for copper-sensing in *S ynechocystis* (at 48 h). InrS *K*_Zn__(__II__)_ (5.6 × 10^−13^ M) is comparable to the sensory sites of ZiaR (and Zur), but Δ*G*_C_^Zn^^(^^II^^)-^^InrS^^·^^DNA^ is less than Δ*G*_C_^Zn(^^II^^)-^^ZiaR^^·^^DNA^ implying that relative to other sensors, Δ*G*_C_^Zn^^(^^II^^)-Sensor·^^DNA^ rather than *K*_Zn__(__II__)_ determines the final detection threshold for Zn(II).

## Introduction

As multiple metalloregulators from different structural families are characterized within a single organism it becomes possible to identify facets of metal homeostasis that result from their concerted actions. Furthermore, as multiple metal sensors from a single family are characterized in different organisms, it becomes possible to confirm (or otherwise) correlations between sequence and structure/function that were inferred from early work. Here, both approaches are taken to develop an appreciation of the factors that determine the metal-selectivity of metalloregulators such as InrS.

*Mycobacterium tuberculosis* CsoR and *Escherichia coli* RcnR are the founder members of the family of metal-responsive repressors that includes *Synechocystis* PCC 6803 (herein referred to as *Synechocystis*) InrS (Iwig *et al*., 2006; 2008; Liu *et al*., 2007; Foster *et al*., 2012). RcnR is a Ni(II) and Co(II) responsive repressor of expression of the RcnA Ni(II) and Co(II) efflux-protein of *E. coli* (Rodrigue *et al*., 2005; Iwig *et al*., 2006; 2008). *E. coli* contains a single paralogue, FrmR, which likely responds to formaldehyde and is not thought to detect metals (Herring and Blattner, 2004). Orthologous NcrB represses expression of Ni(II)-efflux in *Leptospirillum ferriphilum* and responds to Ni(II) (Zhu *et al*., 2011), while InrS itself is a Ni(II)-responsive repressor of the final gene in the *nrs* Ni(II)-efflux operon in the cyanobacterium *Synechocystis* (García-Domínguez *et al*., 2000; Foster *et al*., 2012). CsoR and RicR are paralogues that both respond to Cu(I) in *M. tuberculosis* (Liu *et al*., 2007; Festa *et al*., 2011). *Staphylococcus aureus* also contains CsoR paralogues, one of which detects Cu(I) while the other, CstR, regulates inorganic sulphur metabolism upon reaction with pathway intermediates of sulphate reduction (Baker *et al*., 2011; Grossoehme *et al*., 2011). Cu(I)-sensing CsoR orthologues have also been characterized from *Bacillus subtilis*, *Thermus thermophilus*, *Listeria monocytogenes* and *Streptomyces lividans* (Smaldone and Helmann, 2007; Ma *et al*., 2009a; Sakamoto *et al*., 2010; Corbett *et al*., 2011; Dwarakanath *et al*., 2012).

**Figure 1:**
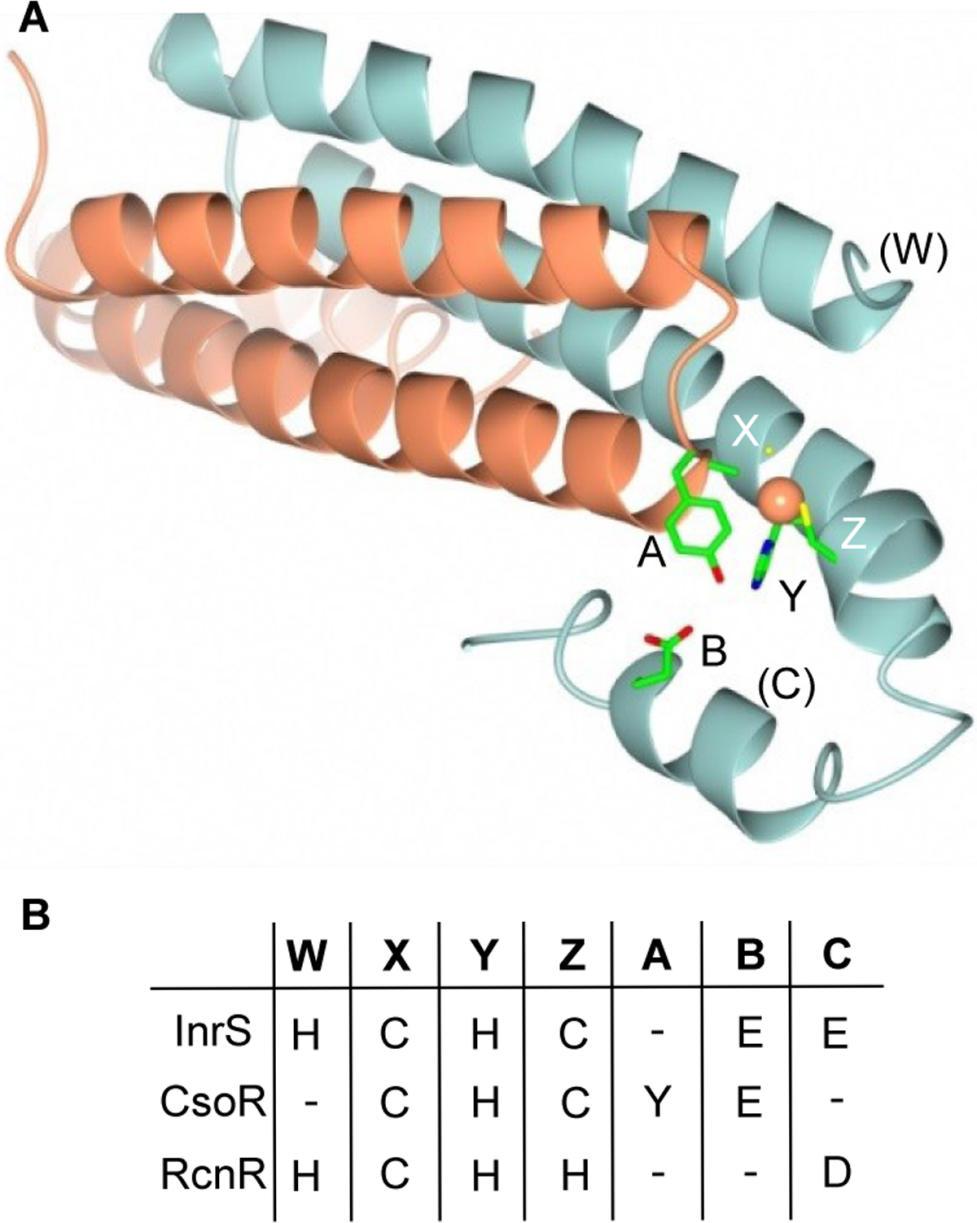
The ‘W-X-Y-Z-(A-B-C)’ fingerprint of CsoR/RcnR family proteins.A. Dimeric representation of *M. tuberculosis* Cu(I)-CsoR (PDB: 2HH7). The side-chains involved in the primary [Cys36′ (X), His61 (Y), Cys65 (Z)] and secondary [Tyr35′ (A) and Glu81 (B)] Cu(I) co-ordination sphere are shown. The approximate location of the ‘W’ position occupied by His3 in *E. coli* RcnR is indicated in parenthesis along with the approximate position of the newly identified ‘C’ position; Cu(I) (orange sphere).B. Residues in the ‘W-X-Y-Z-(A-B-C)’ fingerprint of *M. tuberculosis* CsoR, *E. coli* RcnR and *Synechocystis* InrS. Dash represents lack of a metal-co-ordinating residue in the ‘W’ position, tyrosine in the ‘A’ position, glutamate in the ‘B’ or glutamate/aspartate in the ‘C’ position. There are five histidine residues N-terminal of InrS His21 (W candidate).

RcnR and CsoR bind metal ions at residue positions that partially overlap but the co-ordination numbers and geometries are distinct (Liu *et al*., 2007; Iwig *et al*., 2008). Conserved metal binding residues in this protein family have been used to define a fingerprint to help to predict the function of uncharacterized members (Iwig *et al*., 2008; Ma *et al*., 2009c). *M. tuberculosis* CsoR binds Cu(I) in a trigonal planar site via Cys36′–His61–Cys65 which define the so-called ‘X-Y-Z’ part of the fingerprint (Liu *et al*., 2007) (Fig. S1). The use of ligands located at protomer interfaces creates four symmetry related metal co-ordination sites per tetramer. RcnR retains metal binding residues in the equivalent positions, Cys35′–His60–His64, and additionally RcnR contains His3 which is essential for sensing both Ni(II) and Co(II) *in vivo*, although it may not be obligatory for Ni(II) co-ordination, and defines the ‘W’ position of the fingerprint (Fig. 1) (Iwig *et al*., 2008; Higgins *et al*., 2012). The motif has been extended to include second co-ordination sphere residues that propagate an allosteric response upon Cu(I) binding to CsoR (Ma *et al*., 2009a,b). The Cu(I) complex of CsoR stabilizes hydrogen bonds between the non-co-ordinating face of His61 and second shell Tyr35′ and Glu81 residues that represent the ‘A-B’ part of the fingerprint (Fig. 1) (Liu *et al*., 2007; Ma *et al*., 2009b). RcnR does not conserve the ‘A-B’ motif and moreover His60 is required only for a response to Co(II), not Ni(II), implying a distinct allosteric mechanism relative to CsoR (Iwig *et al*., 2008). In contrast to CsoR, but in common with RcnR, we demonstrate that the allosteric mechanism of InrS does not absolutely require a hydrogen bond network between the ‘A-B-Y’ residues. However, data are consistent with the ‘B’ position Glu98 contributing towards allostery. We identify another conserved glutamate residue in multiple family members lacking the CsoR-like secondary co-ordination sphere fingerprint, and this residue (Glu95 in InrS) also contributes towards allostery defining a new ‘C’ position.

Metal homeostasis in cyanobacteria is of interest due to the status of these organisms as primary producers and ancestors of chloroplasts, coupled with the extreme metal-requirements of the light reactions of photosynthesis and the associated pathways for the assimilation of inorganic elements into organic compounds (Tottey *et al*., 2007). The genome of the model cyanobacterium *Synechocystis* encodes cytosolic metal sensors from four families: MerR activators [including Co(II)-responsive CoaR], Fur co-repressors [including Zn(II)-responsive Zur], ArsR/SmtB de-repressors [including Zn(II)-responsive ZiaR] and a single representative of the CsoR/RcnR family of de-repressors, InrS (Thelwell *et al*., 1998; Rutherford *et al*., 1999; Foster *et al*., 2012; Tottey *et al*., 2012; Patterson *et al*., 2013). The Ni(II) affinity of InrS was previously compared with one representative of each of the other metalloregulatory-families present in this organism and found to be the tightest of the set for this metal. This implies that InrS could be the sole sensor to respond to Ni(II) because it de-represses Ni(II) export via NrsD at concentrations below *K*_Ni(II)_ of the other sensors sustaining a buffered [Ni(II)] sufficiently low to prevent these other proteins from ever gaining access to Ni(II). Precedents where metal specificity of sensing appears to be a function of access to metal *in vivo* include: Failure of NmtR to respond to Ni(II) when transferred from a mycobacterial to a cyanobacterial host that accumulates less Ni(II) (Cavet *et al*., 2002), gain of Mn(II) detection by DtxR when transferred from *Corynebacterium diphtheriae* to *B. subtilis* (Guedon and Helmann, 2003), and acquisition of Mn(II) detection by *B. subtilis* Fur due to mass action when expression is elevated (Ma *et al*., 2012). Preventing aberrant responses to non-effector metals is (arguably) the greater challenge in metal specificity. Here we explore why InrS does not detect copper or Zn(II) *in vivo*, examining whether this is a function of absolute affinity, allostery and/or access, or the relative properties of InrS within the complement of cytosolic metal sensors.

Finally, there is debate as to the physiological significance of the tight metal-binding affinities of many metalloregulators as reported for *E. coli* Cu(I)-CueR and Zn(II)-ZntR for example (Outten and O’Halloran, 2001; Changela *et al*., 2003). The extent to which metal-partitioning to metal sensors approaches thermodynamic equilibrium remains unknown, with recent reports suggesting that ZntR, with estimated *K*_Zn(II)_ 10^−15^ M (Hitomi *et al*., 2001), only responds *in vivo* when the intracellular concentration of Zn(II) rises to 10^−9^ M (Wang *et al*., 2012). By investigating the effects of Zn(II) on DNA binding, by contrasting *K*_Zn(II)_ values of metalloregulatory sites of ZiaR and Zur with InrS, and by monitoring the abundance of ZiaR and InrS regulated transcripts *in vivo*, these arguments are further explored. The possibility that intracellular Zn(II) partitions to metal sensors by associative ligand-exchange reactions involving components of a polydisperse buffer, with no fully hydrated intermediate, is discussed.

## Results

### InrS binds Cu(I) and Zn(II) tightly

InrS-regulated *nrsD* transcripts accumulate after exposure (48 h) to a maximum non-inhibitory concentration of Ni(II) but remain unaltered after analogous treatment with copper or Zn(II) (Foster *et al*., 2012). In theory, InrS could fail to respond to copper and Zn(II) due to a negligible or weak affinity for these metals or an inability to couple the binding of these metals to an allosteric response.

**Figure 2:**
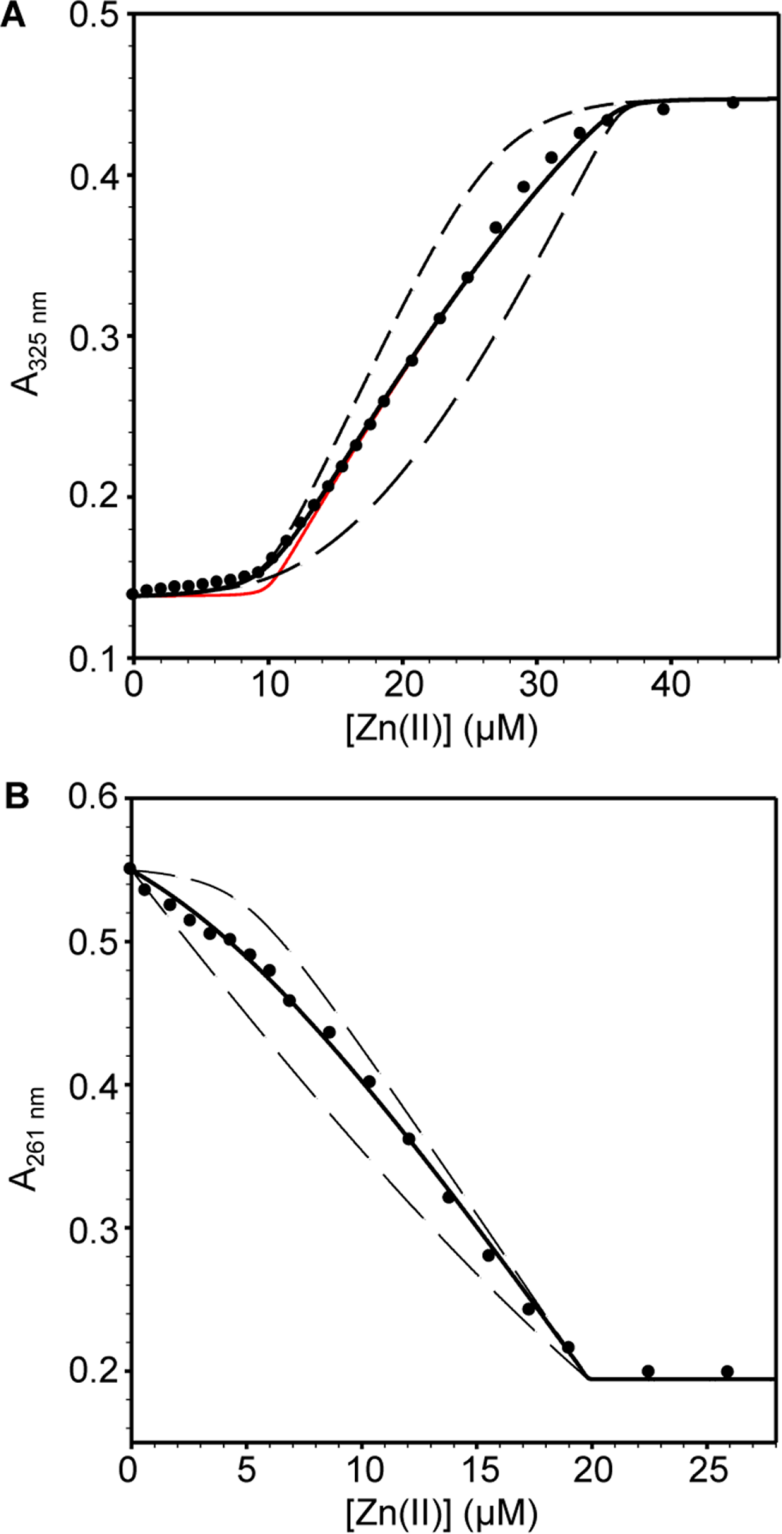
Zn(II) affinity of InrS.A. Representative (*n* = 3) Zn(II)-mag-fura-2 absorbance upon titration of mag-fura-2 (16.2 μM) and InrS (10 μM, protomer) with ZnSO_4_. Solid black line represents fit to a model describing competition from InrS for two molar equivalents of Zn(II) (∴ eight sites per tetramer, with *K*_Zn1–4_ ≪ *K*_Zn5–8_). Solid red line represents a simulated curve with *K*_Zn1–4_ 10-fold tighter than the optimized value and *K*_Zn5–8_ fixed to the optimized value. Dashed lines represent simulated curves with *K*_Zn5–8_ 10-fold tighter and 10-fold weaker than the optimized value and *K*_Zn1–4_ fixed to the optimized value.B. Representative (*n* = 3) quin-2 absorbance upon titration of quin-2 (14.9 μM) and InrS (10 μM, protomer) with ZnSO_4_. Solid line represents fit to a model describing competition from InrS for 0.5 molar equivalents of Zn(II) [first two sites per tetramer (*K*_Zn1–2_)]. Dashed lines represent simulated curves with *K*_Zn1–2_ 10-fold tighter or 10-fold weaker than the optimized value.

The chromophores mag-fura-2 and quin-2 have previously been used to determine the affinity with which metalloproteins bind Zn(II) (VanZile *et al*., 2002; Lisher *et al*., 2013). Both chromophores form 1:1 complexes with Zn(II) with a decrease in the absorbance of quin-2 at 261 nm and an increase in the absorbance of mag-fura-2 at 325 nm reporting on the formation of these complexes (Jefferson *et al*., 1990; Lisher *et al*., 2013). Titration of InrS (10 μM, protomer) and mag-fura-2 [16.2 μM, *K*_Zn(II)_ = 2.0 × 10^−8^ M] with Zn(II) gave a negligible increase in absorbance of mag-fura-2 for the first Zn(II) equivalent (to InrS) implying an affinity substantially tighter than mag-fura-2 (Fig. 2A). Subsequent addition of Zn(II) resulted in an approximately linear increase in absorbance at 325 nm which saturates upon addition of ∼ 36 μM total Zn(II), suggesting that InrS binds a second equivalent of Zn(II) with affinity comparable to that of mag-fura-2. The inferred stoichiometry was confirmed by size exclusion chromatography with buffer supplemented with 20 μM Zn(II) (Fig. S2), where InrS co-migrates with approximately two molar equivalents of Zn(II) [eight Zn(II) ions per tetramer: InrS exists as a tetramer at these concentrations (Foster *et al*., 2012)]. The mag-fura-2 competition data were fit to a model describing one molar equivalent of Zn(II) binding with tight affinity (*K*_Zn1–4_) and a second of weaker affinity (*K*_Zn5–8_), using the fitting software Dynafit (Kuzmic, 1996). This yielded *K*_Zn(II)_ for the weaker sites (*K*_Zn5–8_) of 3.7 (± 0.5) × 10^−8^ M, and the optimized fit departs from simulations with *K*_Zn(II)_ 10-fold tighter or weaker (Fig. 2A). The tightest Zn(II) sites of InrS exceed the limits of the mag-fura-2 competition assay (note red simulated curve on Fig. 2A) and hence quin-2 (*K*_Zn(II)_ = 3.7 × 10^−12^ M) was used to directly measure *K*_Zn(II)_ for the high-affinity sites. InrS (10 μM, protomer) competes with quin-2 (14.9 μM) for 0.5 molar equivalent of Zn(II), namely the first two high-affinity sites per tetramer (*K*_Zn1–2_), the optimized fit gives an affinity of 5.6 (± 2.0) × 10^−13^ M and departs from simulations with *K*_Zn(II)_ 10-fold tighter or weaker (Fig. 2B). All metal binding constants are tabulated (Table 1).

**Table 1:** Zn(II) affinities of InrS, ZiaR and Zur plus Cu(I) affinity of InrS.^a^

	Metal	
Zn(II)	Cu(I)
InrS	*K*_1–2_^b^ = 5.6 (± 2.0) × 10^−13^ M *K*_3–4_^c^ < 2.0 × 10^−8^ M; > 3.7 × 10^−12^ M *K*_5–8_^d^ = 3.7 (± 0.5) × 10^−8^ M	*K*_1–4_^g^ = 7.6 (± 2.8) × 10^−18^ M *K*_5–6_^h^ = 2.8 (± 0.8) × 10^−15^ M
ZiaR	*K*_1–2_^e^ = 4.6 (± 1.7) × 10^−13^ M	–
Zur	*K*_1_^f^ = 2.3 (± 1.9) × 10^−13^ M	–

a Conditions: 10 mM HEPES pH 7.8, 100 mM NaCl, 400 mM KCl for InrS. 10 mM HEPES pH 7.8, 30 mM NaCl, 120 mM KCl for ZiaR and Zur.

b Fit to a model describing Zn(II) binding with equal affinity to the first two sites (*K*_1–2_) on an InrS tetramer, determined by competition with quin-2 (*n* = 3).

c Range represents the fact that sites 3 and 4 (*K*_3–4_) on an InrS tetramer outcompete mag-fura-2 for Zn(II) but fail to compete with quin-2.

d Fit to a model describing Zn(II) binding with equal affinity to the last four sites (*K*_5–8_) on an InrS tetramer, determined by competition with mag-fura-2 (*n* = 3).

e Fit to a model describing Zn(II) binding with equal affinity to the first two sites (*K*_1–2_) on a ZiaR dimer, determined by competition with quin-2 (*n* = 3).

f Fit to a model describing Zn(II) binding to the tightest exchangeable site (*K*_1_) on a Zur dimer, determined by competition with quin-2 (*n* = 3).

g Fit to a model describing Cu(I) binding with equal affinity to the first four sites (*K*_1–4_) on an InrS tetramer, determined by competition with BCS (*n* = 4).

h Fit to a model describing Cu(I) binding with equal affinity to the fifth and sixth sites (*K*_5–6_) on an InrS tetramer, determined by competition with BCS (*n* = 4).

**Figure 3:**
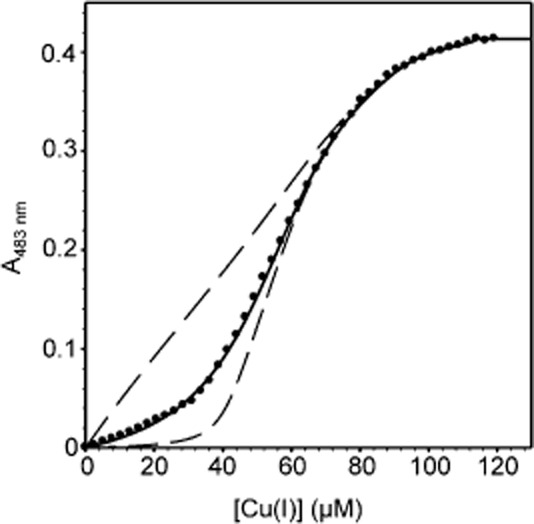
Cu(I) affinity of InrS. Representative (*n* = 4) BCS absorbance upon titration of BCS (68 μM) and InrS (40 μM, protomer) with CuCl [> 95% Cu(I)]. Solid line represents fit to a model describing competition from InrS for two molar equivalents of Cu(I) (∴ eight sites per tetramer, with *K*_Cu__1–4_ ≪ *K*_Cu__5–6_ ≪ *K*_Cu__7–8_). Dashed lines represent simulated curves with *K*_Cu1–4_ 10-fold tighter and 10-fold weaker than the optimized value and *K*_Cu__5–6_ and *K*_Cu__7–8_ fixed to the optimized value.

Bathocuproine disulphonate (BCS) binds Cu(I) with a 2:1 stoichiometry (*β*_2_ = 10^19.8^ M^−2^) and is used to determine Cu(I) affinities of metalloproteins (Xiao *et al*., 2011). Titration of InrS (40 μM, protomer) and BCS (68 μM) with Cu(I) gave a modest increase in the absorbance of BCS at 483 nm (diagnostic of the BCS_2_Cu(I) complex) up to 40 μM (Fig. 3). Subsequent additions of Cu(I) gave greater increases in absorbance at 483 nm with an inflection at ∼ 110 μM total Cu(I), suggesting a Cu(I) binding stoichiometry of eight per tetramer, as observed for Zn(II) and as previously reported for Co(II) (Patterson *et al*., 2013). Using Dynafit the data was fit to a model describing binding of one molar equivalent with tight affinity (*K*_Cu1–4_) followed by half molar equivalents with step-wise weaker affinity (*K*_Cu5–6_ and *K*_Cu7–8_) [eight Cu(I) sites per tetramer]. *K*_Cu1–4_ = 7.6 (± 2.8) × 10^−18^ M, which departs from simulations with *K*_Cu(I)_ 10-fold tighter or weaker (Fig. 3). *K*_Cu5–6_ = 2.8 (± 0.8) × 10^−15^ which also departs from simulations with *K*_Cu(I)_ 10-fold tighter or weaker (Fig. S3A), while the final two sites are too weak for this assay (note simulations on Fig. S3B). Again, *K*_Cu(I)_ values are tabulated (Table 1).

### Cu(I) and Zn(II) dissociate complexes of InrS with the *nrsD* operator-promoter

**Figure 4:**
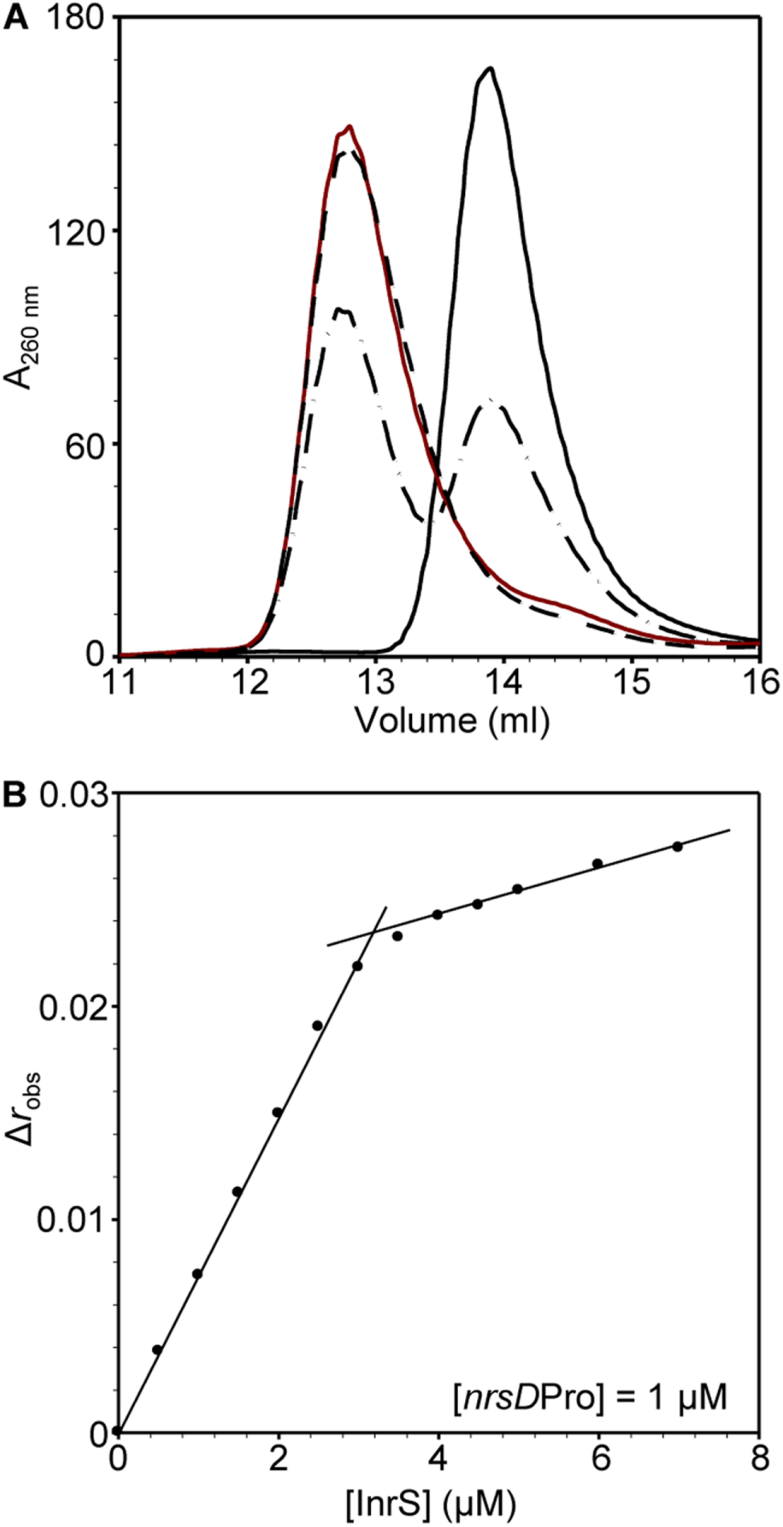
DNA binding stoichiometry of InrS.A. Elution profile obtained from Superdex 75 10/300 GL used to resolve 10 μM unlabelled *nrsD*ProFA pre-incubated with 0 μM (solid black line), 20 μM (dot-dashed line), 40 μM (solid red line) or 80 μM (dashed line) InrS (protomer concentration).B. Anisotropy change upon titration of *nrsD*ProFA (1 μM) with apo-InrS (protomer concentration stated). Experiment performed aerobically in the presence of 1 mM DTT and 5 mM EDTA.

Fluorescence anisotropy was used to monitor the interaction of InrS with a fluorescently labelled fragment of the *nrsD* operator-promoter (*nrsD*ProFA). Ni(II) was previously shown to inhibit interaction between complexes of InrS and *nrsD*ProFA (Foster *et al*., 2012). The degree to which metal binding allosterically inhibits or promotes DNA binding by metalloregulators can be expressed as the coupling free energy Δ*G*_C_, determined from the difference in DNA binding affinity between the apo- and holo-protein forms (Guerra and Giedroc, 2012). For metal-dependent de-repressors such as InrS, a more positive Δ*G*_C_ value indicates greater inhibition of DNA binding upon metal binding. Protein–DNA stoichiometry is required to calculate Δ*G*_C_, and for RcnR and CsoR this is one and two tetramers per operator site respectively (Iwig and Chivers, 2009; Ma *et al*., 2009a,b; Dwarakanath *et al*., 2012; Tan *et al*., 2014). InrS–DNA stoichiometry was determined in two separate types of experiment. Monitoring the co-migration of (unlabelled) *nrsD*ProFA (10 μM) by size exclusion chromatography showed that only 40 μM InrS (protomer) was required to bind all DNA species (Fig. 4A). Similarly, monitoring binding of InrS to a relatively high concentration of *nrsD*ProFA (1 μM) by fluorescence anisotropy revealed a point of inflection at ∼ 4 μM InrS (protomer) (Fig. 4B).

**Figure 5:**
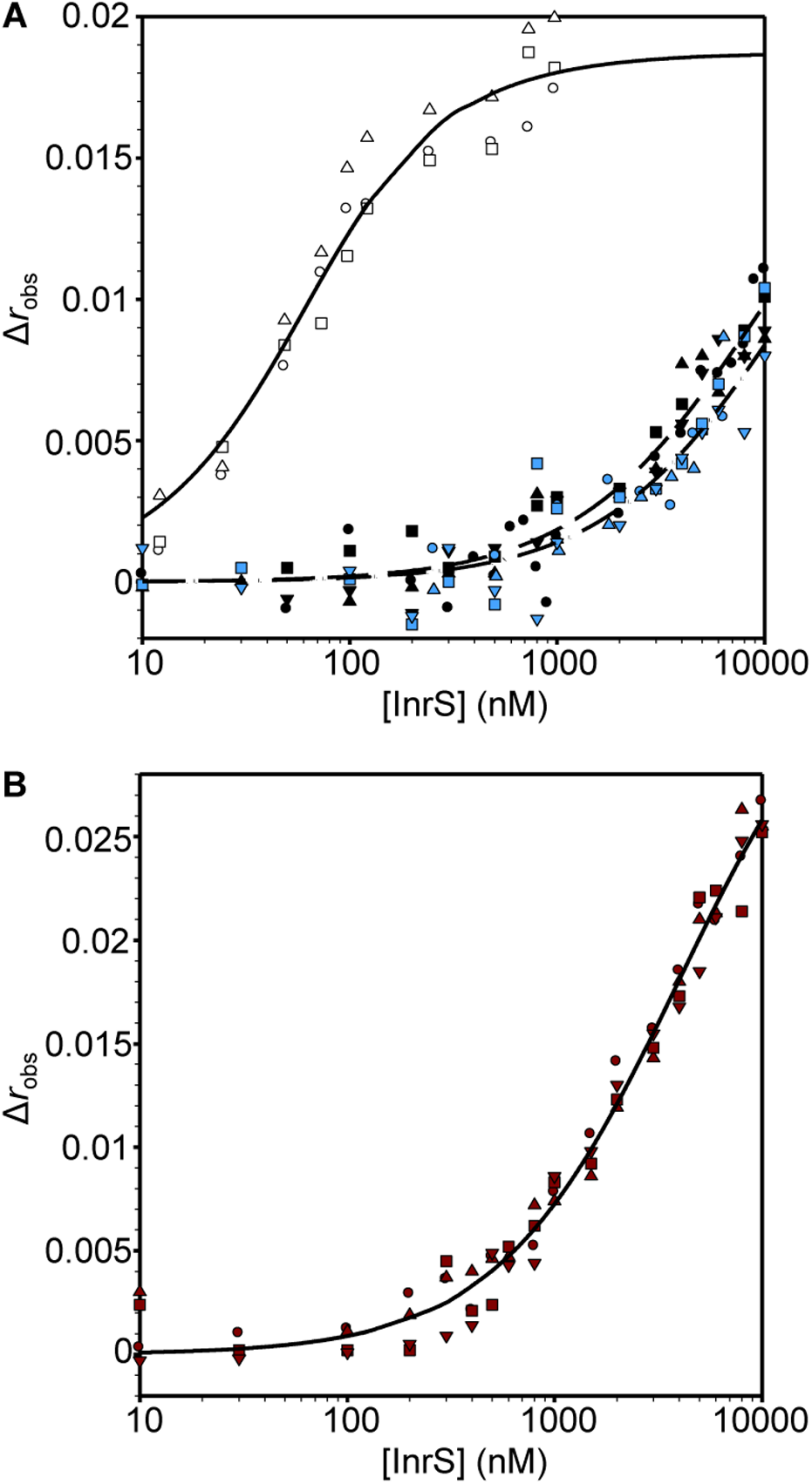
Determination of the DNA binding coupling constant for binding of Ni(II), Cu(I) and Zn(II) to InrS.A. Anisotropy change upon titration of *nrsD*ProFA (10 nM) with either InrS in the presence of 5 mM EDTA (open symbols), Ni(II)-InrS (closed symbols) or Cu(I)-InrS (blue symbols). Symbol shapes represent individual experiments. Data were fit to a model describing a 1:1 InrS tetramer (non-dissociable) : DNA stoichiometry and lines represent simulated curves produced from the average *K*_DNA_ determined across the experiments shown [solid line = apo-InrS, dashed line = Ni(II)-InrS, dot-dashed line = Cu(I)-InrS].B. As ‘A’ with Zn(II)-InrS.

*nrsD*ProFA was titrated with apo-, Ni(II)- and Cu(I)-InrS and anisotropy data fit to a model describing the binding of one non-dissociable InrS tetramer per DNA molecule as noted in Table 2 footnotes (Fig. 5A). The calculated DNA binding affinities (*n* ≥ 3) were converted via the standard thermodynamic function (*Experimental procedures* and footnotes to Table 2) yielding Ni(II)-InrS Δ*G*_C_ = +3.3 (± 0.1) kcal mol^−1^ [Δ*G*_C_^Ni(II)-InrS·DNA^] and Cu(I)-InrS Δ*G*_C_ = +3.4 (± 0.1) kcal mol^−1^ [Δ*G*_C_^Cu(I)-InrS·DNA^]. A similar experiment was used to investigate Δ*G*_C_^Zn(II)-InrS·DNA^ (Fig. 5B). The nature of the interaction is distinct giving greater Δ*r*_obs_ which only just begins to saturate by 10 μM (evident on a linear scale Fig. S4), noting that similar effects were observed with Zn(II)–NmtR–DNA complexes (Reyes-Caballero *et al*., 2011). Data were fit using an analogous model to apo-, Ni(II)- and Cu(I)-InrS, with Δ*r*_obs_ optimized in the fitting, and the calculated DNA binding affinity in Table 2 yields Zn(II)-InrS Δ*G*_C_ = +2.8 (± 0.1) kcal mol^−1^ [Δ*G*_C_^Zn(II)-InrS·DNA^].

**Table 2:** DNA binding affinities^a^^b^ and allosteric coupling free energies^c^ of wild-type and InrS variants plus ZiaR

	Metal	*K*_DNA_^d^^d^ (M)	Δ*G*_C_ (kcal mol^−1^)
Wild-type InrS	apo	9.4 (± 2.0) × 10^−9^	–
	Ni(II)	2.3 (± 0.04) × 10^−6^	+3.3 (± 0.1)
	Cu(I)	3.1 (± 0.5) × 10^−6^	+3.4 (± 0.1)
	Zn(II)	9.8 (± 0.9) × 10^−7^	+2.8 (± 0.1)
	Cu(II)	3.6 (± 0.8) × 10^−6^	+3.5 (± 0.1)
E95A InrS	apo	2.2 (± 0.8) × 10^−8^	–
	Ni(II)	1.4 (± 0.4) × 10^−6^	+2.5 (± 0.2)
E98A InrS	apo	2.1 (± 1.0) × 10^−8^	–
	Ni(II)	1.3 (± 0.3) × 10^−6^	+2.5 (± 0.3)
ZiaR	apo	*K*_1_ = 2.2(± 0.8) × 10^−8^	–
		*K*_2_ = 5.3(± 2.2) × 10^−7^	
	Zn(II)	*K*_1_ ≫ 1.0 × 10^−5^	1st dimer > +3.6^f^
		*K*_2_ ≫ 1.0 × 10^−5^	both dimers > +5.4^f^

a. Determined using fluorescence anisotropy.

b. Conditions: 10 mM HEPES pH 7.0, 60 mM NaCl, 240 mM KCl, 25°C. 5 mM EDTA was included for apo-protein titrations, 1.2 molar excess of metal over InrS and 2.2 molar excess of metal over ZiaR for the metal-loaded titrations.

c. Δ*G*_C_ = −*RT*ln*K*_C_.

d. For InrS (wild-type and variants) represents fit to a model describing one non-dissociable tetramer binding to DNA, with standard deviation (*n* ≥ 3).

e. For ZiaR represents fit to a model describing two dissociable dimers binding to DNA, with standard deviation (*n* = 4). *K*_Dimer_ fixed to 5.0 × 10^6^ M^−1^ by analogy to BxmR (Liu *et al*., 2008).

f. Δ*G*_C_ values calculated considering only the first or both ZiaR dimers binding DNA.

### Cys53, Cys82 and His78 are required for Ni(II) binding to a deduced square planar site

**Figure 6:**
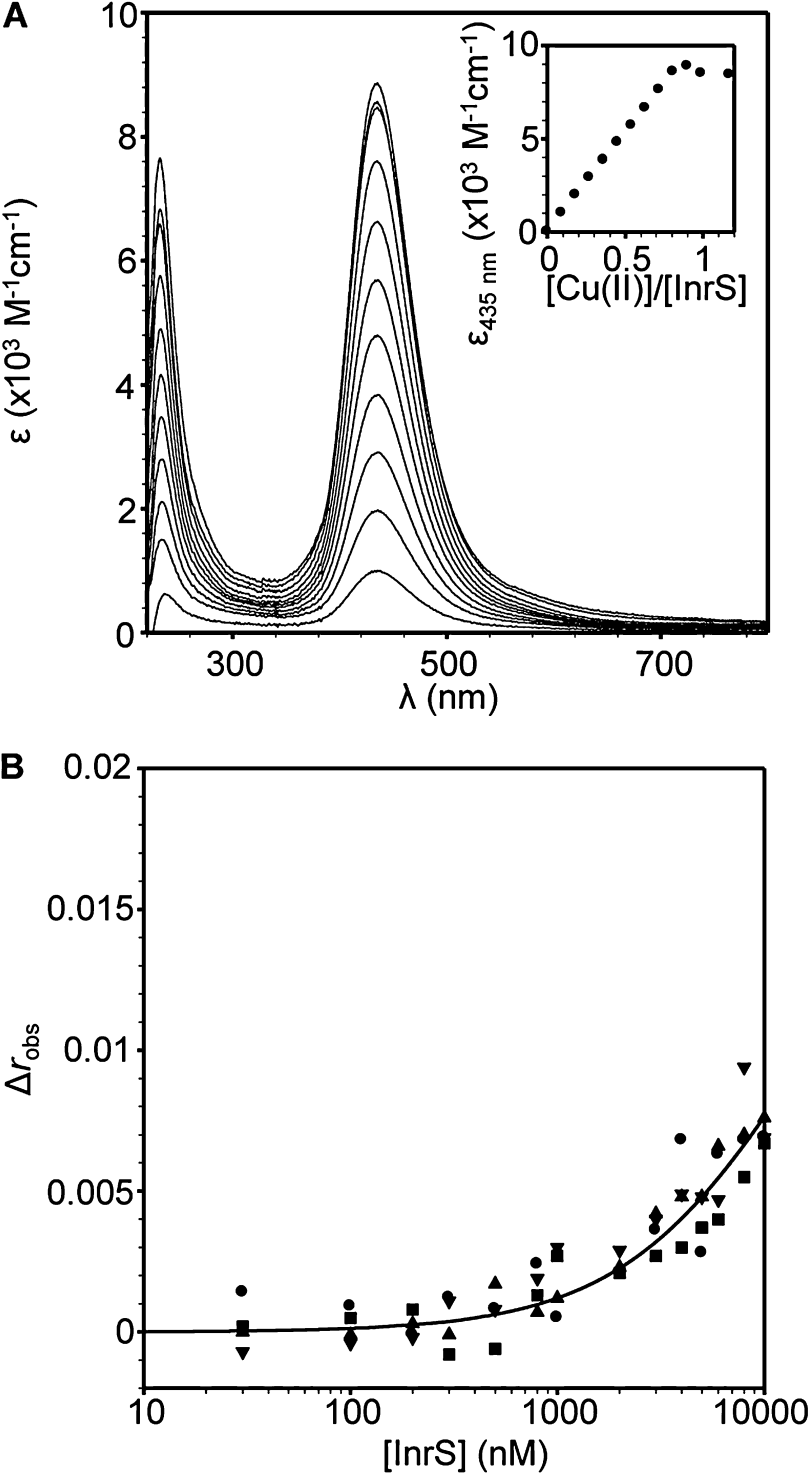
Cu(II) binding properties of InrS.A. UV-vis apo-subtracted difference spectra of InrS (10 μM, protomer) upon titration with CuSO_4_ (pH 7.8). Inset: binding isotherm of the feature at 435 nm.B. Anisotropy change upon titration of *nrsD*ProFA (10 nM) with Cu(II)-InrS, plotted on an equivalent scale to Fig. 5A. Symbol shapes represent individual experiments. Data were fit to a model describing a 1:1 InrS tetramer (non-dissociable) : DNA binding stoichiometry and the solid line represents a simulated curve produced from the average *K*_DNA_ determined across the experiments.

InrS is unlike CsoR in that both Ni(II) and Cu(I) are nearly equally effective at driving the protein from DNA (Fig. 5A, Table 2). RcnR does not respond to copper *in vivo* although it remains unclear whether Cu(I) can disrupt RcnR–DNA complexes *in vitro* (Iwig *et al*., 2008; Higgins *et al*., 2013). It is already known that the spectral properties of Ni(II)-InrS are unlike *E. coli* RcnR and are suggestive of Ni(II) co-ordinated in a square planar geometry (Iwig *et al*., 2008; Foster *et al*., 2012). Cu(II) readily adopts such a geometry. Titration of InrS with Cu(II) results in the formation of an intensely yellow solution due to absorbance at 435 nm (Fig. 6A). The spectra are reminiscent of Cu(II)-NikR where Cu(II) has been shown, crystallographically, to be co-ordinated with a square planar arrangement, and thus lend support to the assignment of a square planar Ni(II) site in InrS (Wang *et al*., 2004; Abraham *et al*., 2006; Phillips *et al*., 2008). Cu(II) also impairs formation of InrS–DNA complexes (Fig. 6B). Anisotropy data were fit to the same model as for Ni(II)-InrS and the calculated DNA binding affinities determine Cu(II)-InrS Δ*G*_C_ = +3.5 (± 0.1) kcal mol^−1^ [Δ*G*_C_^Cu(II)-InrS·DNA^] (Table 2).

**Figure 7:**
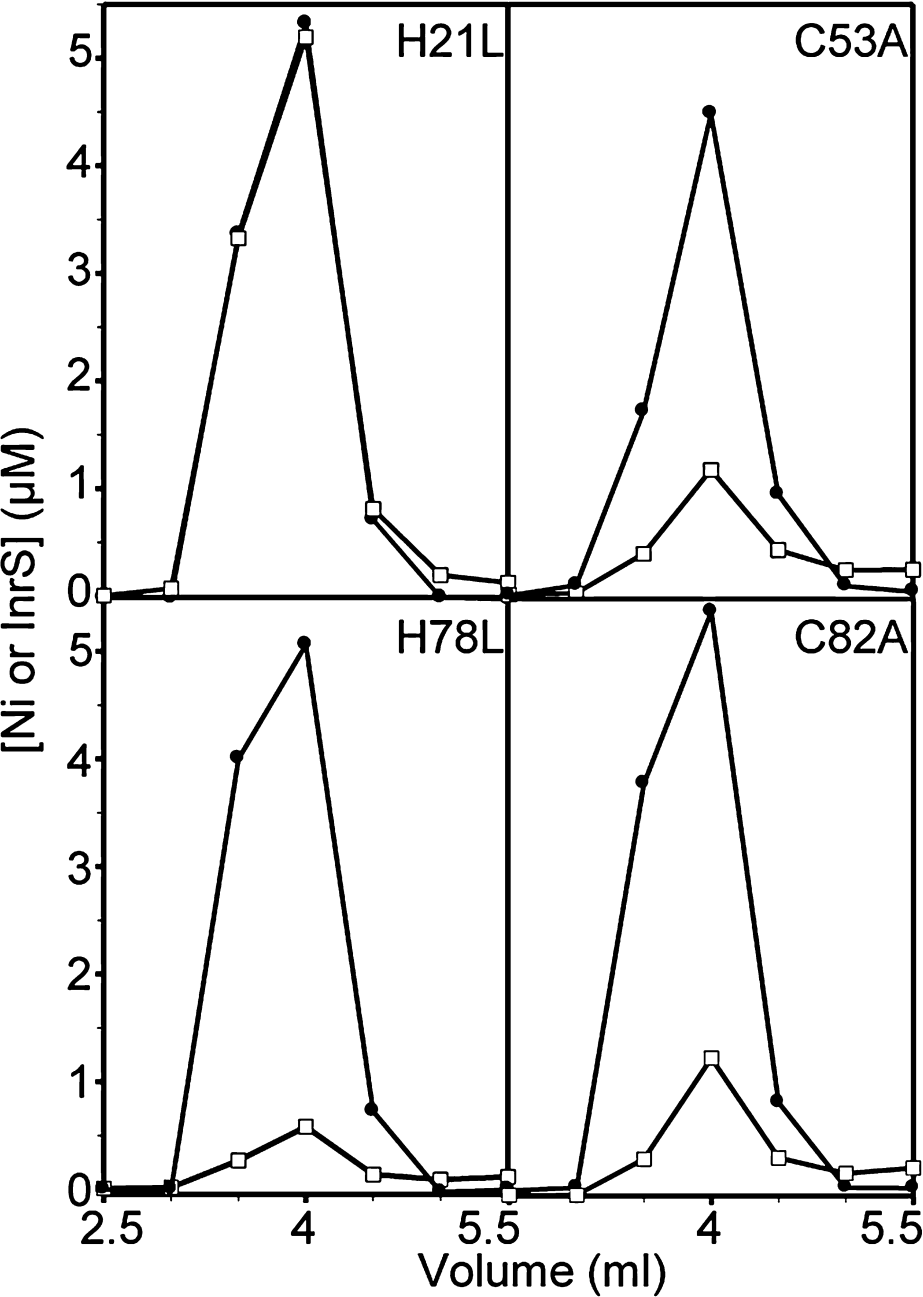
Identification of candidate Ni(II) binding residues. Elution profiles of InrS variants incubated with NiCl_2_ and subjected to size exclusion chromatography. Each variant (10 μM, protomer) was incubated with 14.6 (H21L), 15 (C53A), 19.4 (H78L) or 15 (C82A) μM NiCl_2_ before fractionation on Sephadex G75. Fractions were analysed for protein (solid circles) by Bradford assay and nickel (open squares) by ICP-MS.

Candidate metal-liganding amino acid side-chains found in the ‘W-X-Y-Z’ fingerprint of InrS (His21, Cys53, His78, Cys82) (Fig. 1, Fig. S1), were substituted with non-ligating alternatives. Ni(II) binding was substantially impaired in three cases (C53A, H78L and C82A) but retained in the fourth (H21L) as monitored via co-elution by size exclusion chromatography (Fig. 7). Additionally, three of these mutants (H21L, H78L and C82A) retained the ability to bind Co(II) although with altered UV-vis spectra relative to each other and relative to wild-type InrS (Fig. S5). There was a notable reduction of the ligand to metal charge transfer (LMCT) intensity of C82A consistent with the loss of sulphur from the binding site and a red shift in the *d-d* transition observed for Co(II)-H21L which could be indicative of water occupying an open co-ordination position on Co(II). Residues identical to those forming the tridentate S_2_N site in CsoRs (Liu *et al*., 2007; Ma *et al*., 2009a; Grossoehme *et al*., 2011), are thus all required for Ni(II) co-ordination by InrS, consistent with a primary metal co-ordination sphere which appears (in large part) similar to that of homologous CsoR Cu(I) sensors and unlike the (N/O)_5_S six-co-ordinate site of the related RcnR Ni(II) sensor (Iwig *et al*., 2008). It remains unclear whether or not His21 is a ligand and notable that there are five additional His residues in an extended (relative to RcnR) N-terminal region (Fig. S1).

### The contribution of Glu98 and Glu95 to allostery

**Figure 8:**
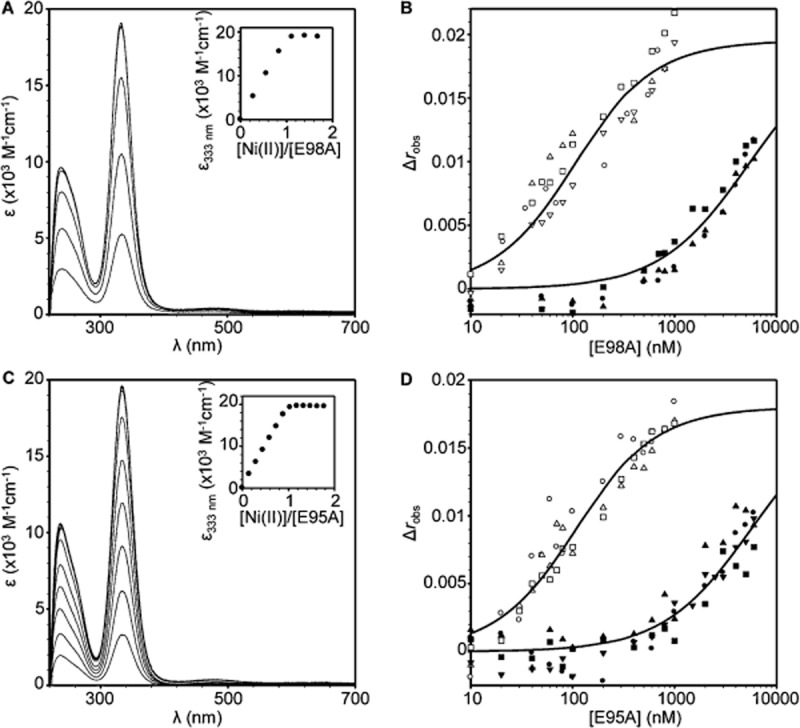
Glu98 and Glu95 contribute to allosteric coupling on Ni(II) binding.A. UV-vis apo-subtracted difference spectra of E98A (6.94 μM, protomer) upon titration with NiCl_2_ (pH 7). Inset: binding isotherm of the feature at 333 nm.B. Anisotropy change upon titration of *nrsD*ProFA (10 nM) with either E98A in the presence of 5 mM EDTA (open symbols) or Ni(II)-E98A (closed symbols). Symbol shapes represent individual experiments. Data were fit to a model describing a 1:1 E98A tetramer (non-dissociable) : DNA stoichiometry and lines represent simulated curves produced from the average *K*_DNA_ determined across the experiments.C. As ‘A’ with E95A (13.1 μM, protomer).D. As ‘B’ with E95A.

InrS conserves histidine (His78) and glutamate (Glu98) at the ‘Y’ and ‘B’ fingerprint positions in common with CsoR but lacks a tyrosine in the ‘A’ position (a proline occupies this position in InrS) (Fig. 1, Fig. S1). In *B. subtilis* and *M. tuberculosis* CsoR the second shell glutamate (‘B’ position) is required to propagate the allosteric response upon Cu(I) binding such that substitution of this residue with alanine reduces Δ*G*_C_^Cu(I)-CsoR·DNA^ from 3.6 to 0.6 kcal mol^−1^ in *B. subtilis* CsoR and from ≥ 5.4 to −0.9 kcal mol^−1^ in *M. tuberculosis* CsoR (Ma *et al*., 2009a,b). The second co-ordination sphere tyrosine appears to tune the magnitude of the response reducing the Δ*G*_C_^Cu(I)-CsoR·DNA^ by approximately one-third when substituted with phenylalanine in *M. tuberculosis* CsoR (Ma *et al*., 2009b), and the equivalent substitution in *L. monocytogenes* CsoR results in an ∼ 50% reduction in copper induction from a CsoR regulated promoter in a β-galactosidase assay (Corbett *et al*., 2011). As His78 is an essential ligand for Ni(II) (Fig. 7), we decided to test if InrS might use a (partially) similar hydrogen bond connection to that in CsoR by making an E98A mutation. E98A shows Ni(II) binding spectra essentially identical to the wild-type protein and it associates with *nrsD*ProFA although *K*_DNA_ is weakened relative to wild-type (Fig. 8A and B, Table 2). Crucially, Ni(II) remains competent to impair the formation of complexes between variant E98A and the *nrsD* operator-promoter with Δ*G*_C_^Ni(II)-E98AInrS·DNA^ = +2.5 (± 0.3) kcal mol^−1^, and *K*_DNA_ for Ni(II)-E98A is tighter than wild-type (Fig. 8B, Table 2). A hydrogen bond involving the first co-ordination sphere His78 and second co-ordination sphere Glu98 is not absolutely required for allostery in InrS. This essential component of the mechanism connecting metal binding to impaired DNA binding thus remains the preserve of Cu(I) sensors, although Glu98 does contribute to allostery in InrS.

All cyanobacterial InrS homologues, with the exception of Q7NE35 (see *Discussion*) contain a conserved glutamate residue aligning with Glu95 of InrS (Fig. S6), potentially suggesting a critical role for this residue. To test if this residue contributes towards allostery an E95A variant was generated and confirmed to bind Ni(II), displaying similar Ni(II)-dependent spectra to wild-type InrS (Fig. 8C).The value of Δ*G*_C_^Ni(II)-E95AInrS·DNA^ = +2.5 (± 0.2) kcal mol^−1^, is less than wild-type InrS, and in common with E98A there is a similar combination of both weakened apo-E95A *K*_DNA_ and tighter Ni(II)-E95A *K*_DNA_ (Fig. 8D, Table 2). Thus, this residue also contributes towards coupling metal binding and DNA binding and was therefore designated ‘C’, an extension of the predictive fingerprint.

### InrS transiently responds to Zn(II) and copper during cellular adaptation to elevated metal

**Figure 9:**
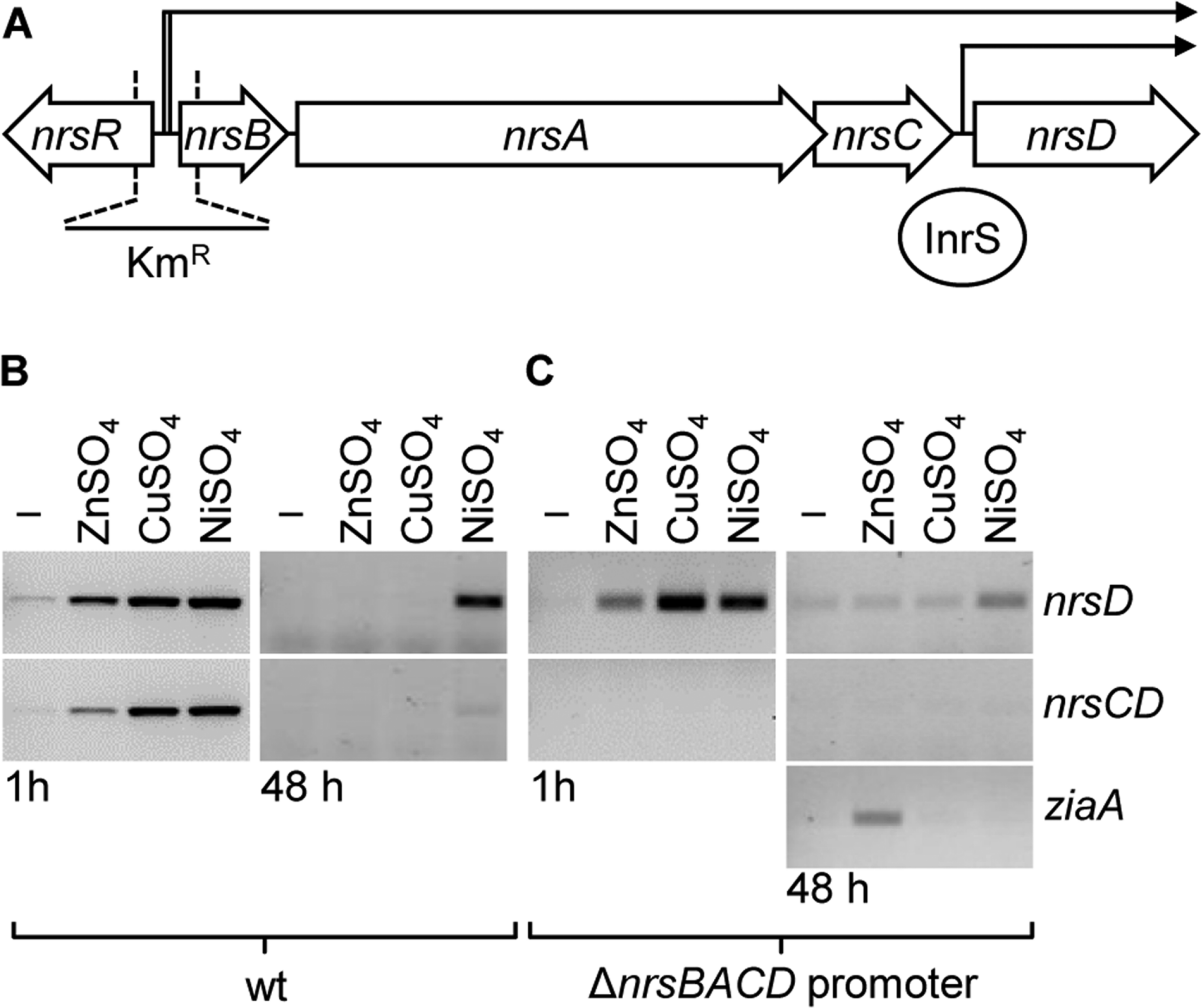
InrS responds transiently to copper and zinc *in vivo*.A. Schematic representation (to scale) of the *nrs* genomic region of *Synechocystis*. The *nrsBACD* promoter region was deleted and replaced by a Km resistance cassette (Km^R^) as indicated.B. *nrsD* and *nrsCD* transcript abundance (by RT-PCR) in wild-type *Synechocystis* cells in response to treatment with maximum non-inhibitory concentrations of NiSO_4_, ZnSO_4_ and CuSO_4_ for 1 h (left) and 48 h (right).C. *nrsD*, *nrsCD* and *ziaA* transcript abundance in Δ*nrsBACD* promoter mutant in response to maximum non-inhibitory concentrations of NiSO_4_, ZnSO_4_ and CuSO_4_ for 1 h (left) and 48 h (right).

InrS is allosterically competent to respond to Cu(I), Cu(II) and Zn(II) *in vitro* (Figs 5 and 6B), yet after 48 h exposure to maximum non-inhibitory concentrations, Ni(II) but not copper or Zn(II), enhanced the abundance of *nrsD* transcripts (Foster *et al*., 2012). One explanation could be that the cell maintains Zn(II) and Cu(I) concentrations below the respective binding constants for InrS. A fluorescent reporter, based upon carbonic anhydrase, indicated that the cytosolic buffered Zn(II) concentration transiently rises to nanomolar levels upon initial (∼ 20 min) exposure of *E. coli* to elevated exogenous zinc (Wang *et al*., 2011). It was reasoned that InrS might transiently respond to exogenous Zn(II) and/or copper if an analogous pulse of elevated [metal] occurred inside *Synechocystis* during adaptation, and indeed *nrsD* transcript abundance increases 1 h after exposure to Zn(II) and copper, but not at 48 h [Fig. 9B, Fig. S7 (loading controls)].

Internal promoters within an operon can be influenced by transcription from upstream promoters and this has recently been shown to confer Zn(II)-responsive repression from promoters located downstream of a Zur-regulated promoter (Napolitano *et al*., 2013). *nrsD* transcripts can be generated both from the InrS regulated *nrsD* operator-promoter and from the NrsR/S-regulated promoter as part of a polycistronic message (Fig. 9A) (García-Domínguez *et al*., 2000; López-Maury *et al*., 2002; Foster *et al*., 2012). Additionally, RNA polymerase and/or the closely following ribosome could dislodge InrS from DNA allowing access to the *nrsD* operator-promoter region (Epshtein and Nudler, 2003; Proshkin *et al*., 2010). Polycistronic transcripts, detected by RT-PCR using primers adjacent to the *nrsCD* intergenic region, accumulated after 1 h exposure to copper and (to a lesser extent) Zn(II) [Fig. 9B, Fig. S7 (loading controls)]. A strain was generated in which the NrsR/S-regulated operator-promoter was removed by homologous recombination-mediated insertion of a kanamycin (Km) resistance cassette (Fig. 9A, Fig. S8). This strain is confirmed to no longer make polycistronic transcripts containing the *nrsCD* intergenic region [Fig. 9C, Fig. S7 (loading controls)], and a smaller increase in abundance of *nrsD* transcripts after 48 h in elevated [Ni(II)] suggests that the ‘dislodging’ of InrS (allowing continuation of transcripts initiated at the NrsR/S-regulated promoter or allowing access to the *nrsD* promoter) may, in part, contribute towards metalloregulation. Importantly in this strain, purely under the control of InrS, *nrsD* transcripts still accumulate in response to Zn(II) and copper but only at 1 h. Thus, InrS is innately promiscuous and can respond to metals other than Ni(II) *in vivo* provided it gains access to sufficiently high metal concentrations. These observations are consistent with the buffered [Zn(II)] transiently exceeding the detection threshold for InrS at 1 h but somehow dropping below this set point by 48 h, presumably due to the actions of homeostatic systems for Zn(II) under the control of *Synechocystis* ZiaR and Zur (Thelwell *et al*., 1998; Tottey *et al*., 2012). Notably, in the same population of transcripts, ZiaR-regulated *ziaA* transcripts accumulate at 48 h [Fig. 9C, Fig. S7 (loading controls)], indicating that Zn(II) must now be maintained at a level that is greater than the detection threshold for ZiaR, but below that required for de-repression by InrS.

### InrS K_Zn__(__II__)_ is similar to *S ynechocystis* Zn(II) sensors ZiaR and Zur

Zn(II) affinities of ZiaR and Zn_1_Zur [purified with one equivalent of Zn(II) kinetically trapped in a structural site (Tottey *et al*., 2012)] were determined by competition with quin-2 (Fig. 10A and B). Zur binds two molar equivalent of Zn(II) per dimer in addition to two structural Zn(II) ions per dimer (Fig. S9), and ZiaR binds four equivalents of Zn(II) per dimer (Dainty *et al*., 2010). Both ZiaR (26.2 μM, protomer) and Zn_1_Zur (20.7 μM, protomer) compete with quin-2 (18.3 and 19.2 μM respectively) for the first molar and first 0.5 molar equivalent of Zn(II) per dimer respectively, reporting on the first two high-affinity sites per dimer for ZiaR (*K*_Zn1–2_) and first for Zn_1_Zur (*K*_Zn1_). Optimized fits give *K*_Zn1–2_ = 4.6 (± 1.7) × 10^−13^ M and *K*_Zn1_ = 2.3 (± 1.9) × 10^−13^ M for ZiaR and Zn_1_Zur respectively, both departing from simulations with *K*_Zn(II)_ 10-fold tighter or weaker (Fig. 10A and B). Metal binding constants are again tabulated (Table 1). Notably for ZiaR, filling of the first two Zn(II) sites per dimer dissociates ZiaR-*zia* operator-promoter DNA complexes (Dainty *et al*., 2010; Tottey *et al*., 2012), and the two tightest Zn(II) sites per tetramer dissociate InrS from DNA (Fig. S10), as with Ni(II) (Foster *et al*., 2012). InrS *K*_Zn(II)_ is thus similar to ZiaR and Zur for Zn(II)-sensing.

**Figure:**
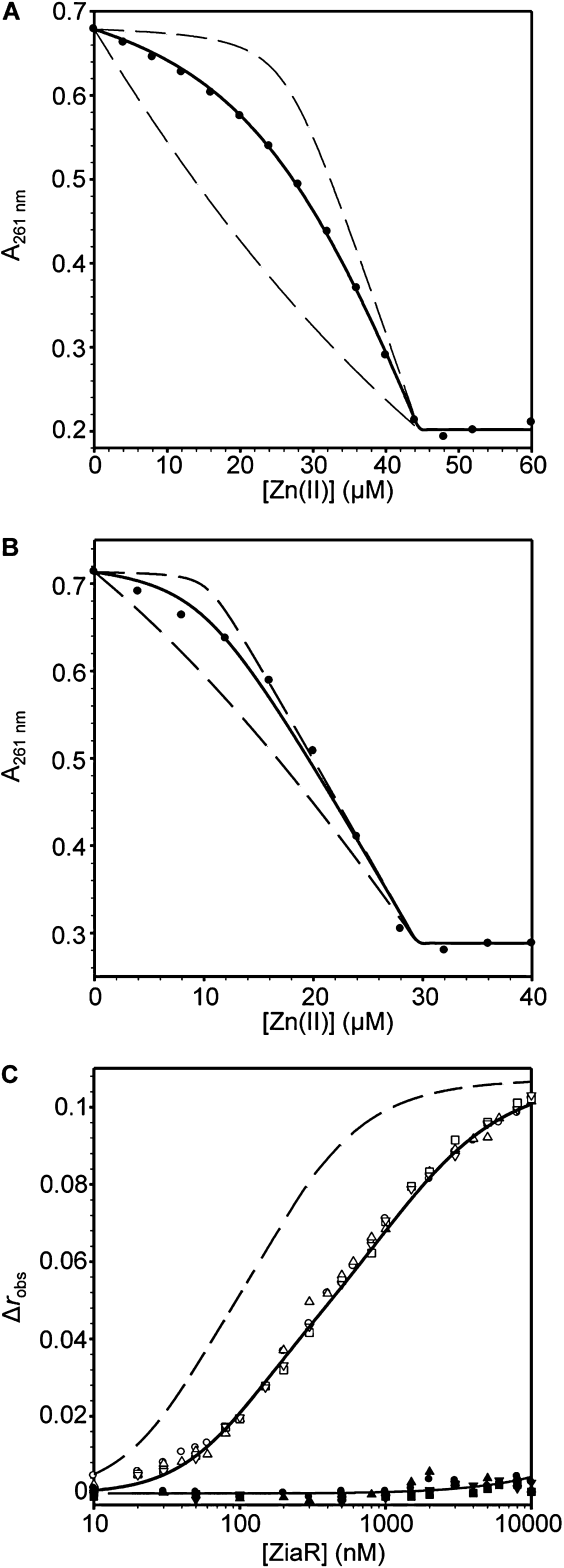
Zn(II) binding properties of ZiaR and Zn_1_Zur.A. Representative (*n* = 3) quin-2 absorbance upon titration of quin-2 (18.3 μM) and ZiaR (26.2 μM, protomer) with ZnCl_2_. Fit to a model describing competition from ZiaR for one molar equivalent of Zn(II) [first two sites per dimer (*K*_Zn1–2_)] (solid line). Simulated curves with *K*_Zn1–2_ 10-fold tighter or 10-fold weaker than the optimized value (dashed lines).B. Representative (*n* = 3) quin-2 absorbance upon titration of quin-2 (19.2 μM) and Zur (20.7 μM, protomer) with ZnCl_2_. Solid line represents fit to a model describing competition from Zur for 0.5 molar equivalents of Zn(II) [first site per dimer (*K*_Zn1_)]. Dashed lines represent simulated curves with *K*_Zn1_ 10-fold tighter or 10-fold weaker than the optimized value.C. Anisotropy change upon titration of fluorescently labelled *zia* o/p DNA (10 nM) with either ZiaR in the presence of 5 mM EDTA (open symbols) or Zn(II)-ZiaR (closed symbols). Symbol shapes represent individual experiments. Data fit to a model describing a 2:1 ZiaR dimer : DNA stoichiometry, with *K*_Dimer_ fixed to 5.0 × 10^6^ M^−1^, and the solid line through the apo data is a simulated curve using the average *K*_DNA_ determined across the experiments. Simulated curve with *K*_DNA_ for both binding events 10-fold tighter than the optimized value to demonstrate the calculated *K*_DNA_ is not limited by the monomer-dimer linkage (dashed line). The solid line for Zn(II)-ZiaR is a simulated curve with *K*_DNA1 and 2_ 20 000-fold weaker than the apo-form. *K*_DNA1 and 2_ may not scale linearly with Zn(II).

### Δ*G*_C_^Zn^^(^^II^^)-^^InrS^^·^^DNA^ is less than Δ*G*_C_^Zn^^(^^II^^)-^^ZiaR^^·^^DNA^

Unlike ZiaR why doesn’t InrS respond to Zn(II) after 48 h if it has comparable Zn(II) affinity and can be triggered by Zn(II)? Fluorescently labelled *zia* operator-promoter was titrated with apo- and Zn(II)-ZiaR and anisotropy data fit to a model describing the binding of two dissociable ZiaR dimers per DNA molecule (as noted in Table 2 footnotes) (Fig. 10C). The DNA binding affinities (*n* = 4) were determined for apo-ZiaR, while for Zn(II)-ZiaR they must be substantially weaker than 10 μM (Table 2). The standard thermodynamic function (*Experimental procedures* and footnotes to Table 2) yields a minimum Zn(II)-ZiaR Δ*G*_C_ = +5.4 kcal mol^−1^ (Δ*G*_C_^Zn(II)-ZiaR·DNA^), or +3.6 kcal mol^−1^ for solely the first ZiaR dimer on DNA. Thus Δ*G*_C_^Zn(II)-ZiaR·DNA^ is substantially greater than Δ*G*_C_^Zn(II)-InrS·DNA^. Relative Δ*G*_C_ may provide an explanation for why InrS does not respond to Zn(II) in adapted (at 48 h) cells.

## Discussion

The Ni(II) sensor InrS binds non-effectors Zn(II) and Cu(I) tightly (Figs 2 and 3), and both metals can disrupt InrS–DNA complexes (Fig. 5), with Δ*G*_C_ for the coupling between Cu(I) binding and DNA binding (Δ*G*_C_^Cu(I)-InrS·DNA^) being similar to that for the *in vivo* effector Ni(II) (Fig. 5, Table 2). Three predicted first co-ordination-shell residues of the canonical ‘W-X-Y-Z-(A-B)’ fingerprint of the CsoR/RcnR family contribute towards high-affinity Ni(II) binding while His21 (one of six histidine residues towards the N-terminus of the protein) is not an essential ligand (Fig. 7). Cu(II) also disrupts InrS–DNA complexes and Cu(II)-InrS spectra are consistent with a square planar geometry (Fig. 6). Residues (His78 and Glu98, ‘Y’ and ‘B’ in the fingerprint) homologous to residues that form one part of the connection between the metal-co-ordination sphere and DNA binding in CsoR, are conserved, but an InrS variant unable to hydrogen bond (E98A) is inducer-responsive, albeit with smaller Δ*G*_C_, and so the allosteric mechanism of InrS must differ from CsoR (Fig. 8B, Table 2). InrS-regulated *nrsD* transcripts transiently accumulate in response to both copper and Zn(II) in the first hour of exposure to maximum non-inhibitory concentrations of metal but after 48 h solely respond to Ni(II) (Fig. 9). InrS *K*_Zn(II)_ is similar to the Zn(II) sensory sites of ZiaR (and Zur) (Figs 2 and 10, Table 1), suggesting that in cells adapted (for 48 h) to elevated Zn(II), selectivity in favour of Zn(II) does not correlate with relative affinity in the set of metal sensors. Importantly, Δ*G*_C_^Zn(II)-ZiaR·DNA^ is substantially greater than Δ*G*_C_^Zn(II)-InrS·DNA^. It is concluded that InrS metal selectivity is a function of multiple factors. Firstly *K*_Ni(II)_ (Foster *et al*., 2012), and Δ*G*_C_^Ni(II)-InrS·DNA^, are sufficient to detect Ni(II), secondly relative Δ*G*_C_ and/or relative *K*_Metal_ within the set of metal sensors disfavours sensing of copper, and finally relative Δ*G*_C_ disfavours sensing of Zn(II).

Metal-co-ordination geometry correlates with the response to cognate metals in *E. coli* RcnR and *B. subtilis* CsoR (Iwig *et al*., 2008; Ma *et al*., 2009a; Higgins *et al*., 2012). An octahedral site in Ni(II)-RcnR appears to include the N-terminal amine-nitrogen, such that insertion of an additional residue at position two (increasing the distance between His3 and the N-terminal amine) encourages four-co-ordinate binding, abrogating Ni(II)-sensing (Iwig *et al*., 2008). In *B. subtilis* CsoR the binding of non-cognate Ni(II) in a square planar geometry results in a much lower Δ*G*_C_ relative to the binding of Cu(I) in the native trigonal planar co-ordination geometry (Ma *et al*., 2009a). Spectral analysis of Ni(II)- (Foster *et al*., 2012), and Cu(II)-InrS (Fig. 6A), suggest that the native co-ordination geometry is square planar, a geometry not adopted by Cu(I) or Zn(II) due to their filled *d* orbitals, and yet binding of the latter metals also drives allostery *in vitro* and (albeit transiently) *in vivo* (Figs 5 and 9). These observations imply that the allosteric mechanism(s) employed by InrS is/are inherently more promiscuous and distinct from either RcnR or CsoR. Ni(II)-dependent co-repressors Nur and NikR have square planar sensory sites while de-repressors RcnR and NmtR have octahedral sites (Cavet *et al*., 2002; Pennella *et al*., 2003; Phillips *et al*., 2008; An *et al*., 2009), suggesting that tight square planar sites in the control of uptake and weaker octahedral sites controlling Ni(II) export, optimize Ni(II) homeostasis (Iwig and Chivers, 2010). InrS, to our knowledge, is the first characterized regulator of Ni(II) export to contain a tight square planar sensory site and is likely the sole cytosolic detector of Ni(II) in *Synechocystis*.

The deduced ‘W-X-Y-Z’ motifs are distinct in founder members CsoR from *M. tuberculosis* and RcnR from *E. coli* (Fig. 1, Fig. S1), suggesting that this might well predict Cu(I) versus Ni(II)-sensing in homologues (Iwig *et al*., 2008; Ma *et al*., 2009c). However, InrS has a cysteine not histidine in the ‘Z’ position (a feature of CsoR), Cu(I)-sensing *T. thermophilus* CsoR the converse pattern (histidine in the ‘Z’ position), the ‘W’ position of *S. aureus* CsoR contains a histidine (a feature of RcnR), while Ni(II)-sensing NcrB is missing histidine in the ‘W’ position (Fig. 1, Fig. S1). Figure 1 shows CsoR/RcnR family members for which the *in vivo* effectors have been determined (black), those encoded by cyanobacterial genomes [blue, plus InrS (Fig. S6)], plus products of *inrS*-like genes proximal to *nrsD*-like genes (grey). The latter are predicted to detect Ni(II) and have a fingerprint analogous to InrS, or missing ‘W’ and thus analogous to CsoR, but none are like RcnR. Thus, the pattern of cysteine versus histidine in the ‘W-X-Y-Z’ fingerprint does not predict which metals are sensed.

**Figure 11:**
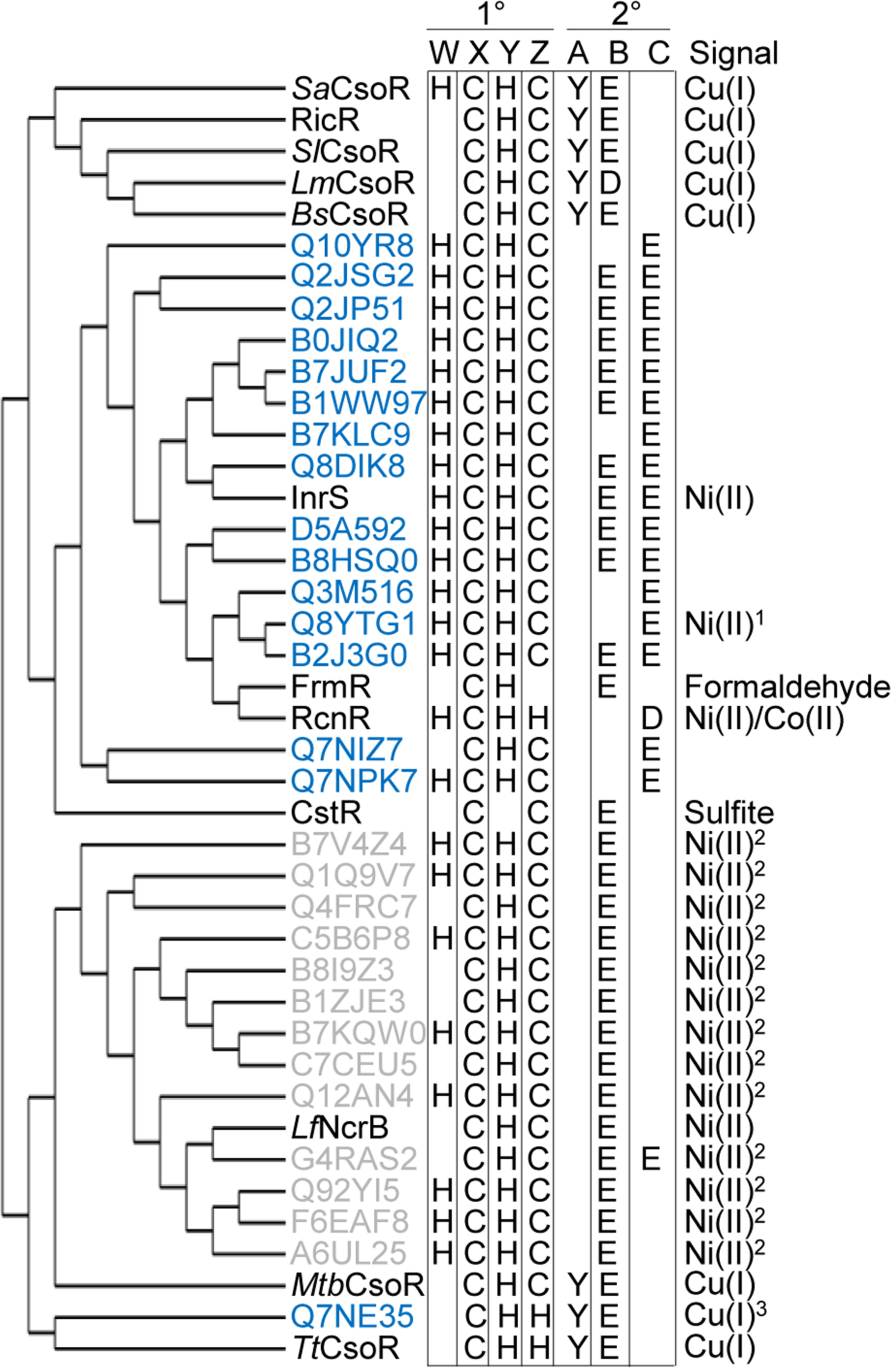
Cladogram and prediction of metals sensed by CsoR/RcnR family proteins. Characterized CsoR/RcnR proteins (black), cyanobacterial InrS-like proteins in organisms listed on Cyanobase (blue) and InrS-like proteins encoded proximal to genes encoding NrsD-like proteins [grey, these were identified previously (Foster *et al*., 2012)]. Uniprot identifiers are used for uncharacterized proteins. Protein sequences were aligned and cysteine or histidine residues in the ‘W-X-Y-Z’ motif positions, tyrosine in the ‘A’ position and either an aspartate or glutamate in the ‘B’ and ‘C’ positions noted. The known or predicted *in vivo* effectors are listed. Predictions are based on: ^1^the discovery of an InrS-like DNA binding site in the promoter region of an *nrsD*-like gene in the organism, ^2^genetic proximity to *nrsD*-like genes, and ^3^genetic proximity to a P-type *ATPase* and predicted copper chaperone encoding genes.

None of the known or deduced Ni(II) sensors in Fig. 1 contain the complete CsoR-like ‘A-B’ (tyrosine-glutamate) fingerprint, and the presence of a second co-ordination sphere tyrosine appears to accurately predict Cu(I) selectivity in this family of proteins. In support of this, the only cyanobacterial homologue to contain a tyrosine in this second co-ordination sphere position (Q7NE35 of *Gloeobacter violaceus* PCC 7421, a primitive cyanobacterium devoid of thylakoids) is predicted to function as a Cu(I) sensor due to its gene context, next to a deduced Cu(I) chaperone and a Cu(I)-translocating P_1_-type ATPase genes. No other cyanobacterial *inrS*-like genes are proximal to known or deduced genes of copper homeostasis but they do contain a conserved glutamate residue (Glu95 in InrS), here designated the ‘C’ position (Fig. 1, Fig. S6). Glutamate is not found at this position in any characterized Cu(I) sensing CsoR and is conspicuous by its absence in Q7NE35. Furthermore, inspection of the 147 curated sequences used to build the hidden Markov model for the Pfam entry of this family (PF02583) reveals that a tyrosine in the ‘A’ position of the fingerprint is always accompanied by a histidine and a glutamate (or aspartate) in the ‘Y’ and ‘B’ positions of the fingerprint respectively, but never by a glutamate or aspartate in this new ‘C’ position that contributes to allostery in InrS (Fig. 8D, Table 2). It is proposed that different connections between the α2 and α3 helices may help to tune metal specificity and that these connections might be a combined function of E95 and E98 in InrS.

Binding of Ni(II) and Cu(I) to InrS results in similar free energies of coupling to DNA binding, whereas binding of Ni(II) to *B. subtilis* CsoR results in a coupling free energy less than a third of that for Cu(I) (Ma *et al*., 2009a). Crucially, Δ*G*_C_^Ni(II)-InrS·DNA^ > Δ*G*_C_^Ni(II)-CsoR·DNA^. The allosteric mechanism of InrS operating for Ni(II) appears to be sufficiently effective (relative to CsoR) to gain a response to Ni(II) *in vivo*. Potentially the dual hydrogen bonds connecting a Cu(I)-liganding histidine to helix α3 mediated by the full ‘AB’ motif in CsoR enables the Δ*G*_C_ term to be sufficient for Cu(I) detection *in vivo* but insufficient for Ni(II) detection. The unknown allosteric connections that operate in InrS do not differentiate between these metals.

As the InrS mechanism can respond to Cu(I) and Zn(II) *in vitro* (Fig. 5, Table 2), how is this avoided *in vivo*? A potential explanation is that the levels of copper and Zn(II) *in vivo* are too low such that the sensor fails to gain access to these elements at sufficient concentrations. This implies that Zn(II) and copper homeostasis operate below the detection threshold for InrS. The detection of surplus copper by *Synechocystis* involves a two-component sensor with no known cytosolic copper sensor (Giner-Lamia *et al*., 2012), and the copper chaperone Atx1 may help withhold copper from InrS (Tottey *et al*., 2012). Elevated cytosolic Zn(II) is detected by ZiaR and Zur (Thelwell *et al*., 1998; Tottey *et al*., 2012), but here we show that *K*_Zn(II)_ for both of these Zn(II) sensors is similar to *K*_Zn(II)_ InrS (Figs 2 and 10, Table 1). Selectivity in favour of Ni(II) correlates with relative affinity within the cells set of metal sensors such that InrS de-represses Ni(II) export at threshold concentrations below *K*_Ni(II)_ of sensors for other metals (Foster *et al*., 2012). An analogous argument cannot be made for Zn(II).

InrS is initially triggered by elevated Zn(II) concentrations, shown by monitoring the abundance of *nrsD* transcripts after 1 h in both wild-type cells and in cells in which the NrsR/S regulated operator-promoter has been inactivated (Fig. 9B and C). After 48 h adaptation, Zn(II)-ZiaR continues to allow detectable de-repression of *ziaA* while apo-InrS now represses *nrsD* (Fig. 9C). How can cells adapt, such that zinc drops below a detection threshold for InrS but not ZiaR, when *K*_Zn(II)_ InrS is similar to *K*_Zn(II)_ ZiaR (Table 1)? Upon formation of Zn(II)-ZiaR, de-repression of *ziaA* leads to increased export of surplus zinc (by ZiaA). The resulting decline in cytosolic Zn(II) concentrations should cause equivalent decreases in fractional Zn(II) occupancies of the sensory sites of ZiaR and InrS (noting that there could also be modulated change in sensor abundance). Because relative Δ*G*_C_ differentiates ZiaR from InrS (Table 2), de-repression of *ziaA* will remain greater than de-repression of *nrsD*, and hence detectable (by RT-PCR), at lower fractional Zn(II)-occupancy: the greater Δ*G*_C_ for Zn(II)-ZiaR compared to Zn(II)-InrS driving the former off-DNA more than the latter.

The buffered cytosolic Zn(II) concentration (after 48 h) would need to fall below ∼ 10^−11^ M (> 10-fold above *K*_Zn(II)_ for InrS, Table 1) for the sensory sites of InrS to become less than saturated with Zn(II). The concentration equating to one free atom per cell-cytosol volume (∼ 1 fl) at any instant is ∼ 10^−9^ M (Outten and O’Halloran, 2001). Thus, the discernment of Zn(II) between InrS and ZiaR in Zn(II)-adapted cells (at 48 h) argues in favour of ZiaR responding to buffered, exchangeable, metal concentrations at least two orders of magnitude below a 10^−9^ M threshold. One (of several possible) scenarios is that a pool of readily exchangeable Zn(II), bound to an excess of relatively weak ligands (amino acids, organic acids, lipids, polypeptides and adventitious ligands on the surfaces of macromolecules including proteins) constituting a polydisperse buffer, partitions to and from metal sensors [and indeed other Zn(II) proteins] via non-specific, ligand-exchange reactions. This associative regime has no requirement for fully hydrated Zn(II) ions and can speed metal exchange towards equilibrium. The discernment of Zn(II) between InrS and ZiaR in Zn(II)-adapted cells (at 48 h) is consistent with such an associative regime, and an associative cell biology for a subset of metals warrants further study.

## Experimental procedures

### Purification of InrS variants, ZiaR and Zur

All proteins were overexpressed, purified and made anaerobic as described previously (Dainty *et al*., 2010; Foster *et al*., 2012; Tottey *et al*., 2012). InrS variants were purified identically to InrS. Protein concentration, reduced thiol content and metal content of anaerobic protein samples were assayed as described previously. All proteins were routinely found to be ≥ 95% metal free and > 90% reduced. All *in vitro* experiments, unless otherwise stated, were carried out under anaerobic conditions using chelex-treated and N_2_-purged buffers.

### Protein–chelator–zinc competitions

Experiments were carried out in 10 mM HEPES pH 7.8, with 100 mM NaCl and 400 mM KCl for InrS and 30 mM NaCl and 120 mM KCl for ZiaR and Zur. ZnSO_4_ (InrS) or ZnCl_2_ (ZiaR and Zur) was titrated into a mixed solution of protein and mag-fura-2 or quin-2 and allowed to equilibrate. Absorbance was recorded at 325 nm (mag-fura-2) or 261 nm (quin-2) using a Perkin Elmer λ35 UV-vis spectrophotometer. Data were fit to the models in the figure legend and Table 1 footnotes using Dynafit to determine Zn(II) binding constants. *K*_Zn(II)_ = 2.0 × 10^−8^ M (determined at pH 7–7.8) for mag-fura-2 (Simons, 1993), and *K*_Zn(II)_ = 3.7 × 10^−12^ M (determined at pH 7) for quin-2 (Jefferson *et al*., 1990). The Zn(II) affinity of mag-fura-2 is independent of pH in the range pH 7–7.8 (Simons, 1993), due to the low *pK*a value of carboxylate ligands (Xiao and Wedd, 2010). The Zn(II) affinity of quin-2, which also utilizes carboxylate ligands, has been reported as near independent of pH in the range pH 7–8 (Reyes-Caballero *et al*., 2010). Mag-fura-2 and quin-2 were quantified using extinction coefficients ε_369nm_ = 22 000 M^−1^ cm^−1^ (Golynskiy *et al*., 2006), and ε_261nm_ = 37 000 M^−1^ cm^−1^ (Jefferson *et al*., 1990) respectively.

### Protein–BCS–Cu(I) competition

CuCl [produced anaerobically from an acidified stock and verified as > 95% Cu(I), as described previously (Dainty *et al*., 2010)], was titrated into a solution of BCS and InrS and the absorbance at 483 nm recorded using a Cary 4E UV-vis spectrophotometer. Data were fit to the model described in the figure legend and Table 1 footnotes using Dynafit with BCS *β*_2, Cu(I)_ = 6.3 × 10^19^ M^−2^ (Xiao *et al*., 2011). Buffer conditions were 10 mM HEPES pH 7.8, 100 mM NaCl, 400 mM KCl. The reported BCS *β*_2, Cu(I)_ is an absolute association constant however due to *pK*a = 5.7 the affinity is essentially invariant at pH > 7 (Xiao *et al*., 2011).

### InrS–DNA co-migration by size exclusion chromatography

Complementary oligonucleotides (21 and 22, Table S1), containing the InrS binding site (identical to those used to create *nrsD*ProFA but unlabelled) were annealed by heating each strand (50 μM) in 10 mM HEPES pH 7.8, 150 mM NaCl to 95°C, then cooling overnight. Annealing was confirmed by native polyacrylamide gel electrophoresis. Under aerobic conditions unlabelled *nrsD*ProFA was incubated with various concentrations of InrS in 10 mM HEPES pH 7.8, 60 mM NaCl, 240 mM KCl, 5 mM EDTA, 2 mM DTT. An aliquot (100 μl) was resolved on Superdex 75 10/300 GL (GE Healthcare) equilibrated in the same buffer.

### Fluorescence anisotropy

InrS variants were prepared in 10 mM HEPES pH 7, 200 mM NaCl, 800 mM KCl with either EDTA (5 mM) or a 1.2 molar excess of CuCl [ > 95% Cu(I)], NiCl_2_, ZnCl_2_ or CuSO_4_ before titrating against fluorescently labelled *nrsD*ProFA [produced as described previously (Foster *et al*., 2012)], in 10 mM HEPES pH 7, 60 mM NaCl, 240 mM KCl with or without 5 mM EDTA depending on whether the InrS variant was apo or metal-loaded. ZiaR was prepared in the same manner using a 2.2 molar excess of ZnCl_2_ where appropriate and titrated against fluorescently labelled *zia* o/p DNA [produced as described previously (Dainty *et al*., 2010)]. Changes in anisotropy were measured using a modified Cary Eclipse Fluorescence Spectrophotometer (Agilent Technologies) fitted with polarizing filters (λ_ex_ = 530 nm, λ_em_ = 570 nm, T = 25°C). Data were fit to the model described in the figure legends and Table 2 footnotes using Dynafit. For Cu(I), Cu(II) and Ni(II) loaded InrS (wild-type and variants) experiments where DNA binding did not saturate the average Δ*r*_obs max_ value from the apo experiments was used in the script. For Zn(II) loaded InrS experiments Δ*r*_obs max_ was optimized during fitting. The coupling free energy Δ*G*_C_, linking DNA binding to metal binding, was calculated via:

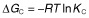
where *R* = 8.314 JK^−1^ mol^−1^ (gas constant), *T* = 298.15 K (temperature at which experiment was conducted), and *K*_C_ = *K*_DNA_^metal^/*K*_DNA_^apo^ (Guerra and Giedroc, 2012), for InrS (1:1 tetramer : DNA stoichiometry) and *K*_C_ = *K*_DNA1_^Zn(II)^·*K*_DNA2_^Zn(II)^/*K*_DNA1_^apo^·*K*_DNA2_^apo^ (Pennella *et al*., 2006), for ZiaR (2:1 dimer : DNA stoichiometry). Mean Δ*G*_C_ values (and S.D.) were calculated from the full set of (equally weighted) possible pair-wise permutations of *K*_C_.

### UV-visible absorption spectroscopy

NiCl_2_ or CuSO_4_ were titrated into a solution of InrS variant in 10 mM HEPES (pH 7 or 7.8 as in text), 100 mM NaCl, 400 mM KCl and the spectra recorded on a Perkin Elmer λ35 UV-vis spectrophotometer.

### Site-directed mutagenesis

Site-directed mutagenesis was carried out via the ‘Quikchange’ method (Stratagene) using pETInrS as template and oligonucleotides 1–12 (Table S1). Following the mutagenesis reaction, methylated template DNA was digested with DpnI. DH5α cells were transformed to kanamycin resistance with the reaction mix and subsequently plasmids were sequenced.

### InrS-Ni(II) co-migration by size exclusion chromatography

InrS variants pre-incubated with NiCl_2_ for 30 min at concentrations stated in figure legends were applied to Sephadex G75 (PD10 column, GE Healthcare) equilibrated with 10 mM HEPES pH 7, 100 mM NaCl, 400 mM KCl then eluted with the same buffer. Fractions were analysed for protein by Bradford assay and for metal by ICP-MS.

### Construction of Δ*nrsBACD* promoter deletion strain

Primers 23 and 24 (Table S1), were used to amplify a 1.8 kb fragment containing the *nrsBACD* promoter and flanking regions from *Synechocystis* genomic DNA and the product ligated to pGEM-T Easy (Promega). Primers 25 and 26 followed by 27 and 28 (Table S1) were used to introduce BamHI restriction sites in the *nrsR* and *nrsB* genes, flanking the *nrsBACD* promoter. Following excision of the *nrsBACD* promoter plus ∼ 115 nt of *nrsR* and *nrsB* using BamHI, a kanamycin (Km) cassette excised from plasmid pUC4K was ligated in its place to create pGEMP*nrsBACD*::Km cassette. EcoRI was used to excise the Km cassette disrupted *nrsBACD* promoter region fragment, which was incubated with wild-type *Synechocystis* to transform the cells to Km resistance. Transformants were selected on BG11 plates (50 μg ml^−1^ Km). Deletion of the *nrsBACD* promoter by insertion of the Km cassette and segregation to all chromosomal copies was confirmed by PCR using primers 29 and 30 (Table S1).

### Isolation of RNA and reverse transcriptase PCR

Logarithmically growing cells were inoculated to OD_800_ = 0.075 in standard BG11 or BG11 supplemented with maximum non-inhibitory concentrations of NiSO_4_ (0.5 μM), ZnSO_4_ (14 μM), or CuSO_4_ (1.0 μM) and cultured for 1 h or 48 h. Total RNA was extracted and cDNA produced as described previously (Foster *et al*., 2012). Reverse transcriptase was omitted from negative controls. Transcript abundance was assessed by PCR with primers 15 and 16 (*nrsD*), 17 and 18 (*nrsCD*), 19 and 20 (*ziaA*), and 13 and 14 (*rps1*, loading control) (Table S1), each pair designed to amplify *∼* 300 bp. *nrsD* expression in wt cells at 48 h was included as a control and performed on a population of transcripts which were distinct from those analysed previously (Foster *et al*., 2012).
