# Supplementary material for: Metal specificity of cyanobacterial nickel-responsive repressor InrS: cells maintain zinc and copper below the detection threshold for InrS
Source: Mol Microbiol. 2014 Apr 14;92(4):797–812. doi: 10.1111/mmi.12594 (PMC4235346; doi:10.1111/mmi.12594)
Supplement: Supplementary file 1 [file mmi0092-0797-SD1.pdf]

Supporting Information

Metal-specificity of cyanobacterial nickel-responsive repressor InrS: Cells maintain zinc and copper below the detection-threshold for InrS

Andrew W. Foster, Rafael Pernil, Carl J. Patterson and Nigel J. Robinson[1]

Department of Chemistry, School of Biological and Biomedical Sciences, University of Durham, DH1 3LE, UK

[1]To whom correspondence should be addressed: nigel.robinson@durham.ac.uk

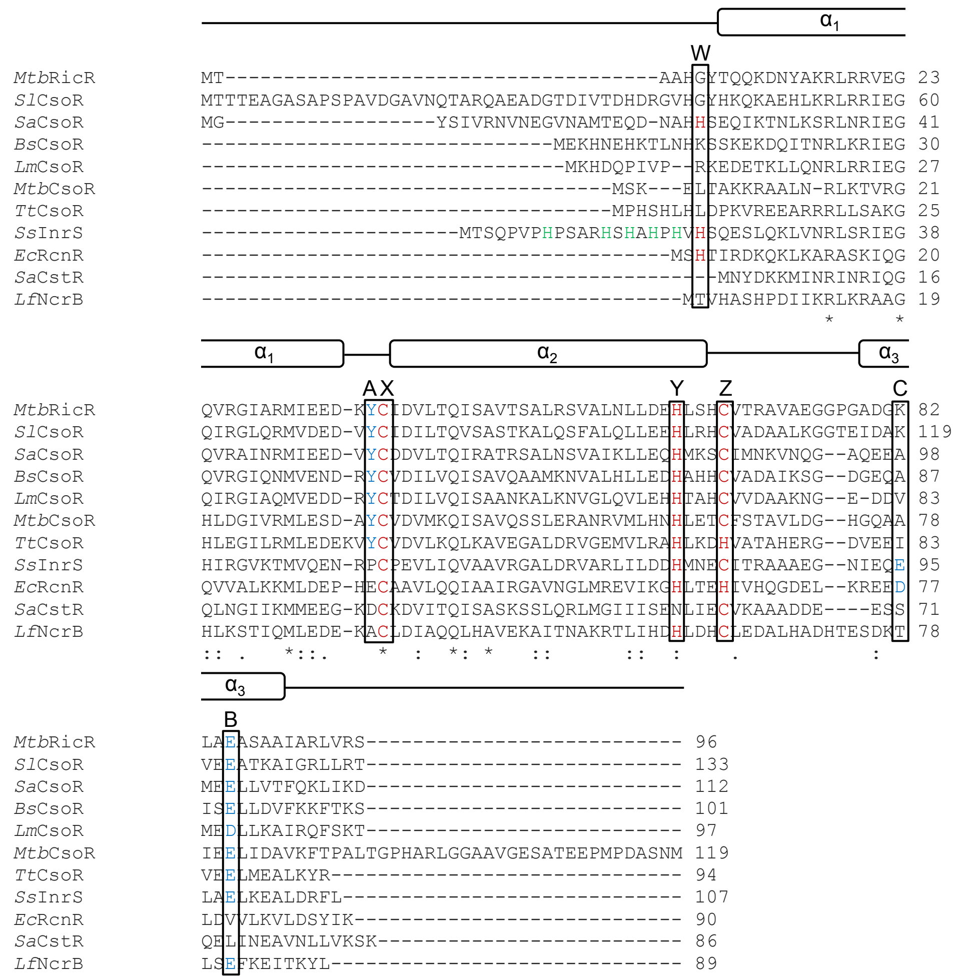

Fig. S1. The 'W-X-Y-Z-(A-B-C)' fingerprint of CsoR/RcnR family proteins. Sequences of CsoR homologues from *Bacillus subtilis* (*Bs*), *Listeria monocytogenes* (*Lm*), *Mycobacterium tuberculosis* (*Mtb*) (CsoR and RicR), *Streptomyces lividans* (*Sl*), *Staphylococcus aureus* (*Sa*), *Thermus thermophillus* (*Tt*), *Escherichia coli* (*Ec*) RcnR, *Synechocystis* (*Ss*) InrS, *Leptospirillum ferriphilum* (*Lf*) NcrB and *Staphylococcus aureus* (*Sa*) CstR. The positions of 'W-X-Y-Z-(A-B-C)' motif residues are indicated and coloured red where there is a metal liganding residue in the predicted primary metal coordination sphere and blue where there are predicted secondary coordination sphere residues analogous to *Mtb*CsoR or where a Glu or Asp residue occupies the 'C' position. The five His residues located N-terminal of InrS His21 are coloured green. Annotated secondary structure features are based on those of *Mtb*CsoR (Liu *et al.*, 2007).

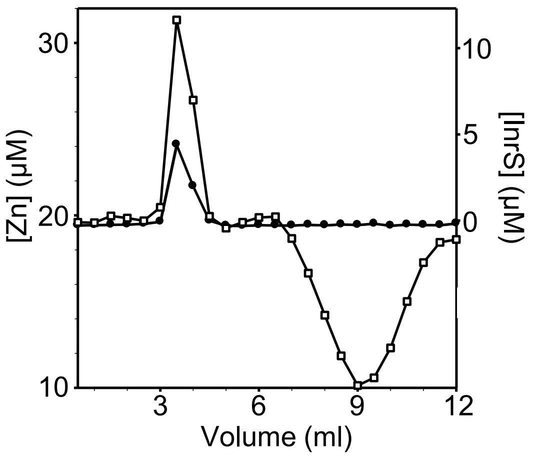

Fig S2. Zn(II) stoichiometry of InrS. Elution profile of Zn(II)-InrS subjected to size exclusion chromatography. InrS (0.5 ml of 10 µM protomer) was applied to Sephadex G75 equilibrated in 10 mM HEPES pH 7.8, 100 mM NaCl, 400 mM KCl, 20 µM $ZnSO_4$ and eluted with the same buffer. Fractions were analysed for protein (solid circles) by Bradford assay and for zinc (open squares) by ICP-MS. Elevated [Zn(II)] in the column buffer system sustains saturation of weak affinity sites.

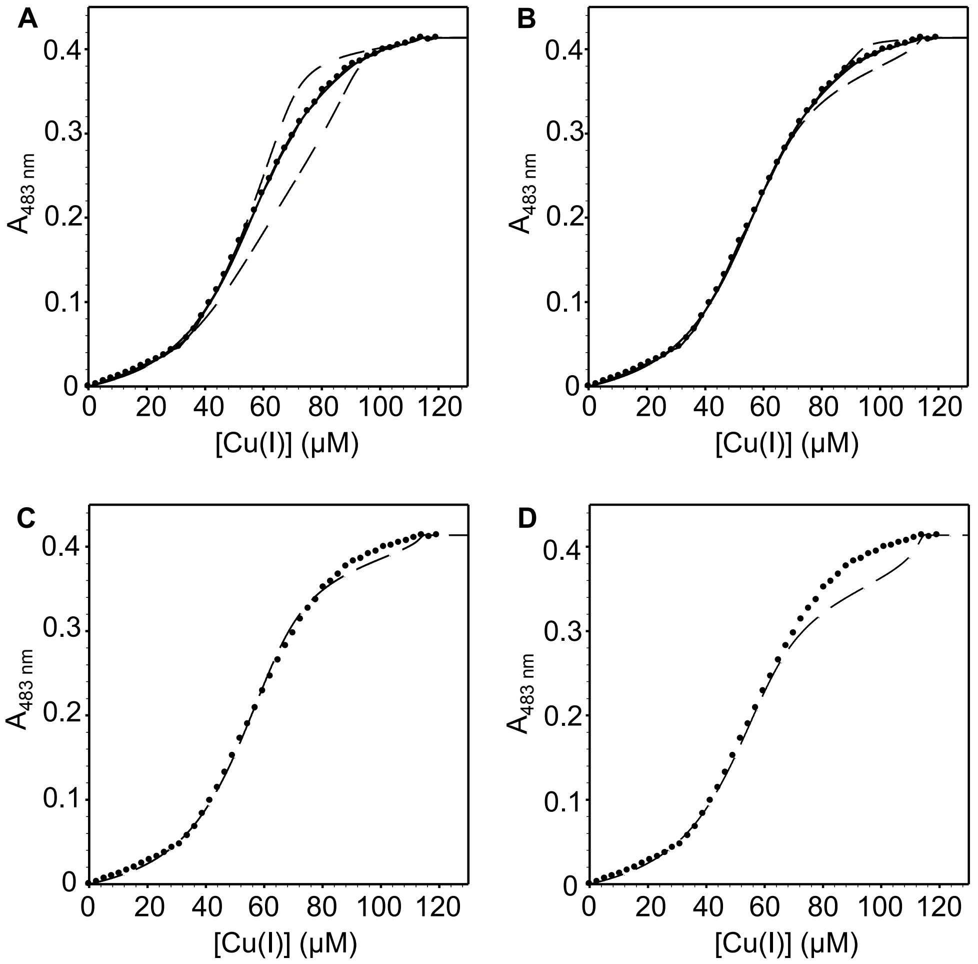

Fig S3. Evaluation of model used to fit data for competition between InrS and BCS for Cu(I). Representative (n = 4) BCS absorbance upon titration of BCS (68 μM) and InrS (40 μM, protomer) (as shown in main text and reproduced here for comparison). Solid line represents fit to the model described in the main text. **A.** Dashed lines represent simulated curves with $K_{Cu5\text{-}6}$ 10-fold tighter and 10-fold weaker than the optimised value and $K_{Cu1\text{-}4}$ and $K_{Cu7\text{-}8}$ fixed to the optimised value. **B.** Dashed lines represent simulated curves with $K_{Cu7\text{-}8}$ 10-fold tighter and 10-fold weaker than the optimised value and $K_{Cu1\text{-}4}$ and $K_{Cu5\text{-}6}$ fixed to the optimised value. These simulated curves suggest that $K_{Cu5\text{-}6}$ can be determined in this experiment but $K_{Cu7\text{-}8}$ cannot. **C.** Dashed line represents fit to a model describing competition from InrS for two molar equivalents of Cu(I) with $K_{Cu1\text{-}4} << K_{Cu5\text{-}8}$. The fit is improved by considering $K_{Cu5\text{-}6}$ separately from $K_{Cu7\text{-}8}$ (Fig. 3). **D.** Dashed line represents a simulated curve with $K_{Cu7\text{-}8}$ fixed to the optimised value for $K_{Cu5\text{-}6}$, with $K_{Cu1\text{-}4}$ and $K_{Cu5\text{-}6}$ fixed to the optimised value.

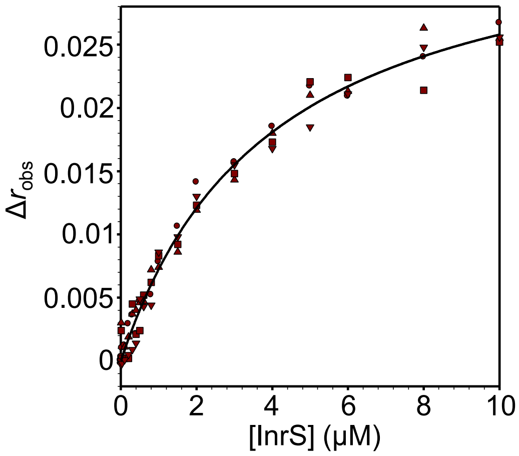

Fig S4. Zn(II)-InrS-DNA complexes display a greater maximum anisotropy value than apo-InrS-DNA complexes. Reproduction of data from Figure 5B, shown here with a linear x-axis for comparison and revealing that the titration is approaching saturation at 10 μM InrS.

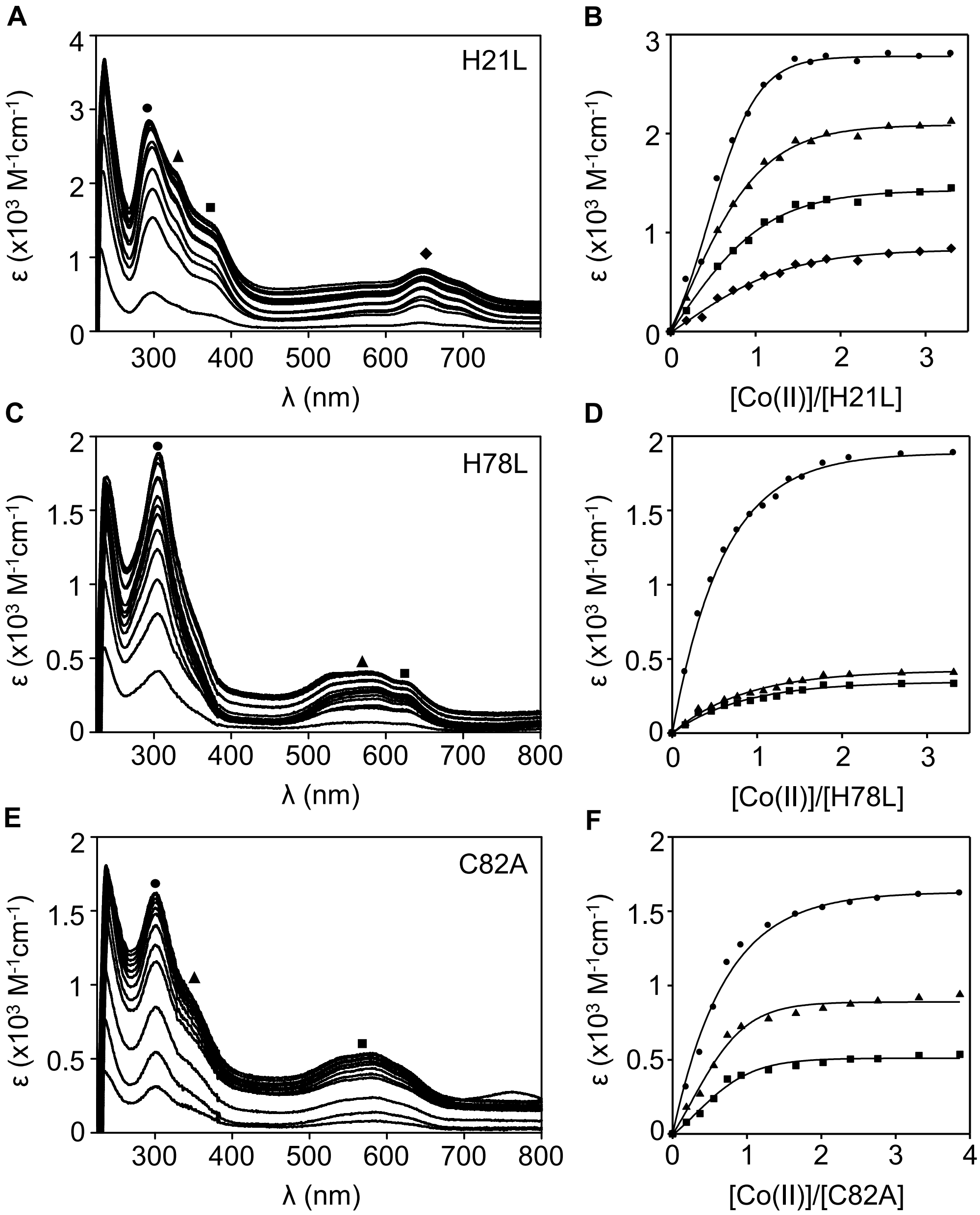

Fig. S5. Apo-subtracted Co(II) dependent UV-vis spectra of InrS variants. A. Titration of H21L (30 μM, protomer) with $CoCl_2$. **B.** Binding isotherms of spectral features shown in '*A*' at 298 nm (circles), 330 nm (triangles), 375 nm (squares) and 650 nm (diamonds). **C.** Titration of H78L (30 μM, protomer) with $CoCl_2$. **D.** Binding isotherms of spectral features shown in '*C*' at 305 nm (circles), 580 nm (triangles) and 628 nm (squares). **E.** Titration of C82A (30 μM, protomer) with $CoCl_2$. **F.** Binding isotherms of spectral features shown in '*E*' at 300 nm (circles), 346 nm (triangles) and 582 nm (squares). Symbols are shown above spectral features to indicate corresponding isotherms. Wild type Co(II)-InrS displays spectral features characteristic of Co(II) bound in a tetrahedral or pseudo-tetrahedral environment with two cysteine ligands (Foster *et al.*, 2012). LMCT spectral features saturated at approximately two molar equivalents of Co(II) and it was suggested this was due to Co(II) being shared between two sites. We have subsequently shown that InrS can bind eight Co(II) ions per tetramer (Patterson *et al.*, 2013). All InrS variant Co(II) dependent spectra shown here are different from those of wild type InrS suggesting that His21, His78 and Cys82 are all ligands for Co(II). C53A displays a broad increase in absorbance with no discrete features, indicative of protein precipitation upon titration with $CoCl_2$. This is suggestive of abrogation of discrete Co(II) binding sites in this mutant and so Cys53 is also suggested as a ligand for Co(II). Experiments performed as described in the main text (pH 7).

```
Q2JP51          ---------------------------------------------------MSDPVPPL- 8
Q2JSG2          -----------------MRGFTKFHISPYCRAPQRSRGKASGIMQTVLCFFMSDPVPPV- 42
Q7NPK7          ---------------------------------------------------MASEVR--- 6
InrS            ------------------------------------------------------------
B8H5Q0          ---------------------------------------------------MVDAISKP- 8
B7KLC9          ---------------------------------------------------MNDQILE-- 7
B0JIQ2          ---------------------------------------------------MTNQITP-- 7
B1WW97          ---------------------------------------------------MFHYLSKPF 9
B7JUF2          ---------------------------------------------------MTTYKDIN- 8
Q8YTG1          ---------------------------------------------------MNGSDQLA- 8
Q3M516          ---------------------------------------------------MNRSNQLA- 8
B2J3G0          ---------------------------------------------------MNGSNRLG- 8
D5A592          ---------------------------------------------------MIGSD---- 5
Q8DIK8          ------------------------------------------------------------
Q10YR8          ---------------------------------------------------MVISNKQK- 8
Q7NE35          ---------------------------------------------------MSKKAVQG- 8
Q7NIZ7          MPSDLASRAALDLSADVIARVFSIDLIEQKIFEILRPDLLLDVLQENLHLDFAGPALLIG 60

                                                         W
Q2JP51          ---RSHSVPPLPSEPDWEEEWELALEKPPLSGR---AQPHHHDPQSRR----------KL 52
Q2JSG2          ---PTGSVPPLPAEPDWEEEWELALERPPLSGR---AQPHHHDPQSRR----------KL 86
Q7NPK7          --------PSLPTSP---------------TG-----TTHTHDPVHTK----------VL 28
InrS            -------MTSQP-------VPHPS--ARHSH-----AHPHVHSQESLQ----------KL 29
B8H5Q0          ----EQELTPSP-------LGAPSSLAAHSHPH---AHPHVHSEESLR----------AI 44
B7KLC9          ----QNKTLNQPSAKK--SHQGTHSHPHDSTGT---PHAHVHTEASLR----------QI 48
B0JIQ2          ----FDPLHN---------HDETG-HDHESKGT---P--HVHSEASLK----------QI 38
B1WW97          ---TVKLLSNIEVKSKTLTQAKIEPTHNHNHGA---SHKHVHSEASLK----------QI 53
B7JUF2          -----ETITNFPLDPS-LEDTHIENHHHHHGGH---P--HIHSEESLK----------KI 47
Q8YTG1          ----QESLPVPQKVEESHNHDAEHDHKHHTHGQAESVHAHVHSEESLR----------RI 54
Q3M516          ----QESLPVPQKVEESHNHDAEHDHKHHTHGQAESVHAHVHSEESLR----------RI 54
B2J3G0          ----KESLPISQQAEHSQHHDTEHEHTDHTHGTGEPAHPHVHSEESLR----------RI 54
D5A592          ----SDRIPS---VSVSDRHHGDASNHSHSH-----PHPHVHSDESLR----------RI 43
Q8DIK8          ---------------MSTESHSHFSPSVHSH-------PHHHSEESLR----------AI 28
Q10YR8          -----KQIGSHT-----LEKDIISEDHHHSS-----AHPHVHSEESLK----------RL 43
Q7NE35          -----------------------------------ATHHLCMPVESRK----------QA 23
Q7NIZ7          NFLEAFHLCACPLCRLYHRRASLSLSRKRGRRGPTIGVPPGSTSGPLRGLLMPHKYTQDV 120
                                                               :

                                       AX                        Y   Z
Q2JP51          TNRLARIEGHVRGIRSMIEQ-DQPCPDVLLQIAAVKGALDRVARLILDDHIRHCIRHAIE 111
Q2JSG2          IHRLARIEGHVRGIRSMIEQ-DQPCPDVLLQIAAVKGALDRVARLILDDHIRHCIRHAIE 145
Q7NPK7          LDRLARIEGHVRGIGNMVRE-DRPCPDVLVQISAVRAALNQVAKLVLKEHLSDCVVHAVE 87
InrS            VNRLSRIEGHIRGVKTMVQE-NRPCPEVLIQVAAVRGALDRVARLILDDHMNECITRAAA 88
B8H5Q0          VNRLSRIEGHIRGIKTMVQE-SRACPDVLVQIAAVRGALDRVARMILDAHLTECIARAAE 103
B7KLC9          INRLSRIEGHIRGIKTMVQE-SRPCPEVLVQIAAVRGAIDRVARIILDEHLSECIARAAE 107
B0JIQ2          INRLSRIEGHIRGIKTMVSE-NRPCPEVLMQIAAIRGAIDRVARMILDEHLSECIARAAQ 97
B1WW97          INRLSRIEGHIRGVKTMVSE-SRPCPEVLIQLAAVRGAIDRVSRLILDEHLNECVARAAQ 112
B7JUF2          TNRLSRIEGHIRGVKTMVTE-NRACPEVLVQLAAVRGAIDRVSRLILDEHLSECIARAAK 106
Q8YTG1          VNRLSRIEGHIRGIKTMVQQ-NSPCPDVLLQIAAVRGALDKVARIVLDEHLTECIARASK 113
Q3M516          VNRLSRIEGHIRGIKTMVQQ-NSPCPDVLLQIAAVRGALDKVARIVLDEHLTECIARASK 113
B2J3G0          ANRLSRIEGHVRGIKTMVQQ-NSPCPDVLLQIAAVRGALDKVARIVLDEHLTECIARAAH 113
D5A592          INRLSRIEGHIRGVKNMVQE-SRPCPDVLVQIAAVRGALDRVARIILDEHLTECIARAAK 102
Q8DIK8          VNRLSRIEGHVRGIKTMVQD-SRPCPEVLIQIAAVRGALDRVARLILDEHLDECVTRAAQ 87
Q10YR8          INRLSRIEGHVRGIKTMIKD-HRACPDVLIQIAAVRGALDRVARIVLDNHLSDCIVRANQ 102
Q7NE35          ARRLAIAKGHLESIQKMLDDPEIYCIDVLRQLKAVQGALSRAGEVVLRGHLEAHVTTAHE 83
Q7NIZ7          LKHLASIEGRVRDVYTTVEA-GGACPDVLVRMVYLRSAVNAAAQLVLKDHTEHCLVEAAG 179
                  :*:  :*::..: . :      * :** ::  ::.*:. ...::*  *    :  *

                      C  B
Q2JP51          TGNIEVELEELQRALDRFIS 131
Q2JSG2          SGNIEVELEELQRALDRYIS 165
Q7NPK7          NGGAHEEIQALNQAIDRYID 107
InrS            EGNIEQELAELKEALDRFL- 107
B8H5Q0          AGNIEAEIEELKTALDRFLN 123
B7KLC9          EGNIDEEIEQLKAALDRFLP 127
B0JIQ2          EGSIETEIEELKAALDRFLP 117
B1WW97          EGNIEEELQELKAALDRFLP 132
B7JUF2          EGNIDAEIEELKAALDRFLP 126
Q8YTG1          EGNIEEEIKQLKAALDRFLP 133
Q3M516          EGNIAEEIEQLKAALDRFLP 133
B2J3G0          EGNIDVEIKELKAALDRFLP 133
D5A592          EGNIDVEIEELKAALDRFLP 122
Q8DIK8          EGRIDQELAELKAALDHFLG 107
Q10YR8          DGDIDVEIAALKSALDRFLP 122
Q7NE35          RGDQGRIVEELMEALKYH-- 101
Q7NIZ7          SERYEAELENFLAAVDMLL- 198
                       :  :  *:.
```

Fig. S6. Multiple sequence alignment of cyanobacterial representatives of the CsoR/RcnR family. The position of the 'W-X-Y-Z-(A-B-C)' motif residues are indicated and coloured red where there is a metal liganding residue in the predicted primary metal coordination sphere and blue where there are predicted secondary coordination sphere residues analogous to *Mtb*CsoR or where there is a Glu or Asp residue in the newly identified 'C' position. The N-terminal regions of many of these proteins are histidine-rich (green). Sequences (Uniprot identifiers shown) are from organisms listed on Cyanobase.

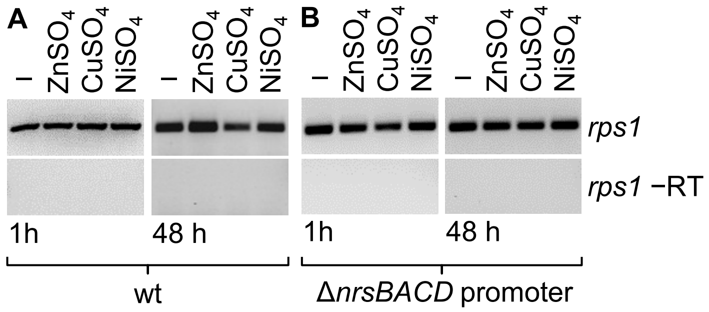

Fig. S7. RT-PCR loading controls. A. RT-PCR analysis of *rps1* transcript abundance in wild type *Synechocystis* transcript populations used in Fig. 10B. **B.** RT-PCR analysis of *rps1* transcript abundance in Δ*nrsBACD* promoter *Synechocystis* transcript populations used in Fig. 10C. –RT indicates that samples that have not been treated with reverse transcriptase and confirm the lack of contaminating DNA.

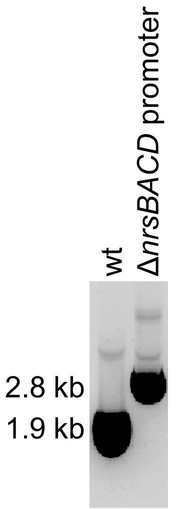

Fig. S8. Δ*nrsBACD* promoter mutant integration and segration to all chromosomal copies. Deletion of the *nrsBACD* promoter by insertion of the kanamycin resistance cassette, and segregation to all chromosomal copies, was confirmed by PCR using primers 29 and 30 (Table S1) to identify a diagnostic 2.8 kb fragment (wild type (wt) gives a 1.9 kb fragment).

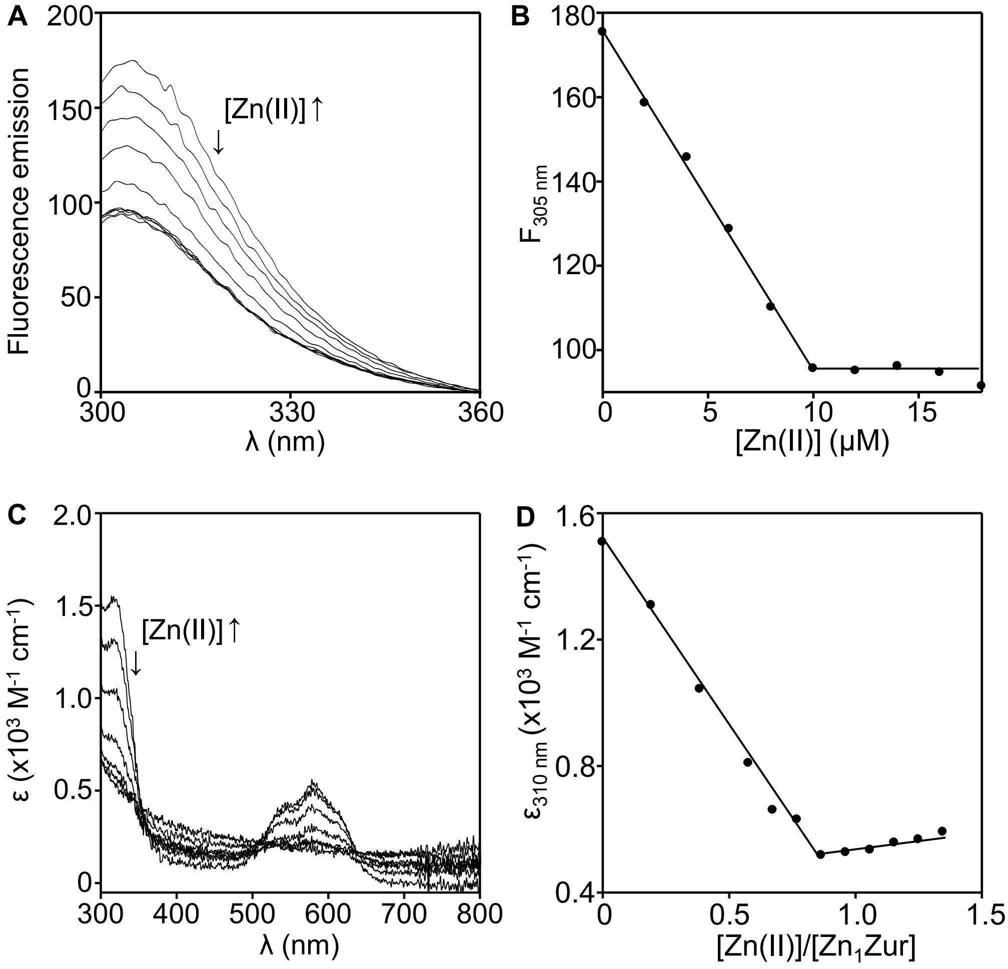

Fig. S9. Zn(II) binding stoichiometry of *Synechocystis* Zn_1Zur. A. Fluorescence emission spectra of Zn_1Zur (*Synechocystis* Zur routinely co-purifies with ~ one molar equivalent of zinc (Tottey *et al.*, 2012)) (10 μM, protomer) upon excitation at 280 nm following titration with $ZnSO_4$. **B.** Binding isotherm depicting the decrease in intensity of the fluorescence emission feature at 305 nm observed in '*A*'. Fluorescence is fully quenched upon addition of one molar equivalent of Zn(II). Data collected using a Cary Eclipse Fluorescence Spectrophotometer (Varian). **C.** Apo-subtracted UV-vis spectra of Co_1Zn_1Zur (26 μM (protomer), $CoCl_2$ present at a 1.1 molar excess) upon titration with $ZnSO_4$. Co(II) dependent spectral features are quenched indicative of displacement by Zn(II). **D.** Binding isotherm depicting the decrease in intensity of the 310 nm LMCT feature observed in '*C*'. The decrease in the intensity of this feature shows an inflection upon the addition of 0.87 molar equivalents of Zn(II). Data collected using a Cary 4E UV-vis spectrophotometer. These data are consistent with a Zn(II) binding stoichiometry of two per dimer in addition to the structural zinc ions. For both experiments anaerobic protein samples were prepared as described in the main text and buffer conditions were 10 mM HEPES pH 7.8,100 mM NaCl, 400 mM KCl.

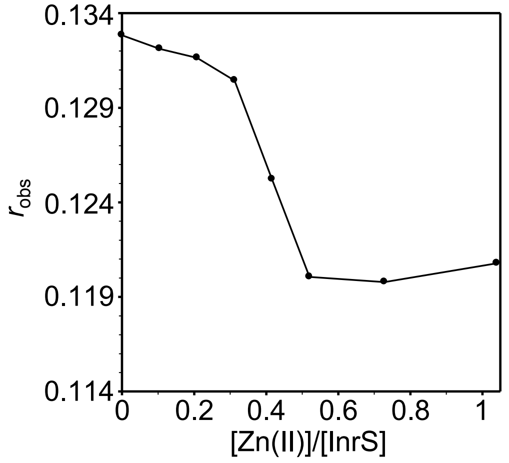

Fig. S10. Filling of two sites per tetramer with Zn(II) drives InrS from DNA. Anisotropy change upon titration of pre-formed *nrsD*ProFA (10 nM) and InrS (1 μM, protomer) with $ZnSO_4$. Data were collected under the conditions described in the main text for anisotropy experiments (pH 7.8). The smaller reduction in r_{obs} relative to that observed in analogous experiments with Ni(II) (Foster *et al.*, 2012) is reflective of the smaller coupling free energy upon Zn(II) binding relative to Ni(II) (Fig. 5, Table 2).

Table S1. Oligonucleotides used in this study.

	Use	Sequence
1	Quikchange H21L	5′-GCCCATCCCCATGTCCTGAGCCAAGAATCCTTAC-3′
2	Quikchange H21L	5′-GTAAGGATTCTTGGCTCAGGACATGGGGATGGGC-3′
3	Quikchange C53A	5′-GCAGGAAAATCGTCCCGCCCCAGAGGTGTTAATTC-3′
4	Quikchange C53A	5′-GAATTAACACCTCTGGGGCGGGACGATTTTCCTGC-3′
5	Quikchange H78L	5′-GATTAATTTTGGATGACCTGATGAATGAGTGCATCACCAG-3′
6	Quikchange H78L	5′-CTGGTGATGCACTCATTCATCAGGTCATCCAAAATTAATC-3′
7	Quikchange C82A	5′-GACCACATGAATGAGGCCATCACCAGGGCGGCG-3′
8	Quikchange C82A	5′-CGCCGCCCTGGTGATGGCCTCATTCATGTGGTC-3′
9	Quikchange E98A	5′-GAGCAGGAGTTGGCGGCGTTGAAGGAAGCCCTAG-3′
10	Quikchange E98A	5′-CTAGGGCTTCCTTCAACGCCGCCAACTCCTGCTC-3′
11	Quikchange E95A	5′-GGCAATATTGAGCAGGCGTTGGCGGAGTTGAAGG-3′
12	Quikchange E95A	5′-CCTTCAACTCCGCCAACGCCTGCTCAATATTGCC-3′
13	*rps1* RT-PCR	5′-CTCTGATTGACATTGGGGCG-3′
14	*rps1* RT-PCR	5′-GAGCGCTGATGTGGGAGCCG-3′
15	*nrsD* RT-PCR	5′-CCCAAGCCATTAGTTTGCTGG-3′
16	*nrsD* RT-PCR	5′-TAAAAAAGGCGTTAAACACATTCAGC-3′
17	*nrsCD* RT-PCR	5′-GGACTTTTCCACCCAATTACAAC-3′
18	*nrsCD* RT-PCR	5′-CGCAAGCAGTGAAAGATAGGC-3′
19	*ziaA* RT-PCR	5′-TGAAGCGCAATGGTACAGTGC-3′
20	*ziaA* RT-PCR	5′-GGGATGAACAGAGGTGGTAAC-3′
21	Production of *nrsD*ProFA	5′-TCATCAATATCCCCCCTGGGGGCATAGAATAGA-3′
22	Production of *nrsD*ProFA	5′-TCTATTCTATGCCCCCAGGGGGGATATTGATGA-3′
23	Cloning *nrsBACD* promoter	5′-CCACTTCTCGCTCCAACG-3′
24	Cloning *nrsBACD* promoter	5′-GGAGCTTCGGTGTGAATATCC-3′
25	Introduction of BamHI site	5′-GATTTTCCAAAGGATCCCAAGCTTGGGAGCC-3′
26	Introduction of BamHI site	5′-GGCTCCCAAGCTTGGGATCCTTTGGAAAATC-3′
27	Introduction of BamHI site	5′-GAGTTTCAAGCCGAGGGATCCATTAATCGGGTTG-3′
28	Introduction of BamHI site	5′-CAACCCGATTAATGGATCCCTCGGCTTGAAACTC-3′
29	Verification of Δ*nrsBACD* promoter	5′-CTAAACTGTCATGCAACGTCC-3′
30	Verification of Δ*nrsBACD* promoter	5′-CTTCTTCCGGTGCCAGAC-3′

Supporting information references

Foster, A.W., Patterson, C.J., Pernil, R., Hess, C.R., and Robinson, N.J. (2012) Cytosolic Ni(II) sensor in cyanobacterium: Nickel detection follows nickel affinity across four families of metal sensors. *J Biol Chem* **287:** 12142-12151.

Liu, T., Ramesh, A., Ma, Z., Ward, S.K., Zhang, L., George, G.N., *et al*. (2007) CsoR is a novel *Mycobacterium tuberculosis* copper-sensing transcriptional regulator. *Nat Chem Biol* **3:** 60-68.

Patterson, C.J., Pernil, R., Dainty, S.J., Chakrabarti, B., Henry, C.E., Money, V.A., *et al*. (2013) Co(II)-detection does not follow $K_{Co(II)}$ gradient: Channeling in Co(II)-sensing. *Metallomics* **5:** 352-362.

Tottey, S., Patterson, C.J., Banci, L., Bertini, I., Felli, I.C., Pavelkova, A., *et al*. (2012) A cyanobacterial metallochaperone inhibits deleterious side effects of copper. *Proc Natl Acad Sci USA* **109:** 95-100.
